# Variation in parent–offspring kinship in socially monogamous systems with extra‐pair reproduction and inbreeding

**DOI:** 10.1111/evo.12953

**Published:** 2016-06-01

**Authors:** Jane M. Reid, Greta Bocedi, Pirmin Nietlisbach, A. Bradley Duthie, Matthew E. Wolak, Elizabeth A. Gow, Peter Arcese

**Affiliations:** ^1^ Institute of Biological and Environmental Sciences, School of Biological Sciences, Zoology BuildingUniversity of AberdeenTillydrone AvenueAberdeenAB24 2TZScotland; ^2^ Department of Evolutionary Biology and Environmental StudiesUniversity of ZurichWinterthurerstrasse 1908057ZurichSwitzerland; ^3^ Department of Forest and Conservation Sciences, 2424 Main MallUniversity of British ColumbiaVancouver BCCanadaV6T 1Z4

**Keywords:** Cuckoldry, paternal investment, paternity, pedigree, polyandry, relatedness, sexual conflict

## Abstract

Female extra‐pair reproduction in socially monogamous systems is predicted to cause cuckolded socially‐paired males to conditionally reduce paternal care, causing selection against extra‐pair reproduction and underlying polyandry. However, existing models and empirical studies have not explicitly considered that cuckolded males might be related to their socially‐paired female and/or to her extra‐pair mate, and therefore be related to extra‐pair offspring that they did not sire but could rear. Selection against paternal care, and hence against extra‐pair reproduction, might then be weakened. We derive metrics that quantify allele‐sharing between within‐pair and extra‐pair offspring and their mother and her socially‐paired male in terms of coefficients of kinship and inbreeding. We use song sparrow (*Melospiza melodia*) paternity and pedigree data to quantify these metrics, and thereby quantify the joint effects of extra‐pair reproduction and inbreeding on a brood's total allelic value to its socially‐paired parents. Cuckolded male song sparrows were almost always detectably related to extra‐pair offspring they reared. Consequently, although brood allelic value decreased substantially following female extra‐pair reproduction, this decrease was reduced by within‐pair and extra‐pair reproduction among relatives. Such complex variation in kinship within nuclear families should be incorporated into models considering coevolutionary dynamics of extra‐pair reproduction, parental care, and inbreeding.

Identifying components of negative and positive selection that shape the evolution and persistence of extra‐pair reproduction in socially monogamous systems remains a central challenge in evolutionary ecology (Arnqvist and Kirkpatrick 2005; Parker and Birkhead 2013; Reid et al. 2015a). In systems with biparental care, it is widely predicted that cuckolded socially‐paired males, who have lost the paternity of extra‐pair offspring (EPO) produced by their socially‐paired female, should under some circumstances reduce their provision of costly paternal care rather than invest in broods that contain unrelated EPO that they did not sire (Sheldon 2002; Kokko and Jennions 2008; Alonzo 2010; Alonzo and Klug 2012; Griffin et al. 2013). Mean rates of extra‐pair reproduction and paternal care might consequently coevolve, and hence be negatively correlated across species (Kokko and Jennions 2008; Griffin et al. 2013; Remeš et al. 2015). Furthermore, paternal care might be reduced through behavioral plasticity within or among males when individuals expect to gain higher reproductive value from future breeding attempts than from investing in a current brood in which paternity has been lost (Westneat and Sherman 1993; Houston and McNamara 2002; Sheldon 2002; Alonzo and Klug 2012). The form and magnitude of such plasticity is predicted to be complex, and to depend on male assessment of paternity and on multiple dimensions of within‐ and among‐individual life‐history variation that shape current versus future reproductive value (Westneat and Sherman 1993; Kokko 1999; Houston and McNamara 2002; Holen and Johnstone 2007; Eliassen and Kokko 2008; Alonzo 2010; Benowitz et al. 2013). However, any reduction in paternal care that occurs following any degree of female extra‐pair reproduction, and that reduces the fitness of the female's offspring, will impose negative selection against extra‐pair reproduction (Arnqvist and Kirkpatrick 2005). Other components of positive direct or indirect selection are then required to maintain female extra‐pair reproduction and underlying polyandry (Kokko 1999; Jennions and Petrie 2000; Arnqvist and Kirkpatrick 2005; Reid et al. 2015a), thereby driving further evolution of male responses to postcopulatory sexual selection, to individual and population‐wide levels of extra‐pair paternity and hence paternity uncertainty, and to emerging conflicts over parental care (Parker et al. 2002; Houston et al. 2005; Kokko and Jennions 2008; Alonzo 2010; Remeš et al. 2015).

One potential source of positive selection on female extra‐pair reproduction, which could counteract any negative selection stemming from reduced paternal care, stems from inbreeding avoidance. Females that initially pair with closely related males are widely hypothesized to produce more EPO, sired by less closely related extra‐pair males (Jennions and Petrie 2000; Kempenaers 2007; Arct et al. 2015; Reid et al. 2015a). Polyandrous females could thereby produce less inbred offspring, reducing expression of inbreeding depression and increasing offspring fitness. However, it is not widely noted that it is somewhat contradictory to hypothesize that any selection against female extra‐pair reproduction that arises because females’ cuckolded socially‐paired males reduce paternal care for unrelated EPO could be balanced by positive selection stemming from reduced inbreeding depression in females’ EPO. This is because, for extra‐pair reproduction to reduce the degree to which a female's offspring are inbred, the female must be related to her socially‐paired male. The socially‐paired male must therefore be related to the female's EPO that he did not sire, but for which he could care. Furthermore, the socially‐paired male could potentially be related to his paired female's extra‐pair male, and hence be related to her EPO through paternal as well as maternal links. Overall, socially‐paired males could then be closely related to EPO that they did not sire but could rear.

However, to date, verbal hypotheses and analyses of models that examine the degree to which cuckolded males should reduce paternal care for broods of offspring produced by their socially‐paired females typically assume that cuckolded males are unrelated to the female's EPO, and have not explicitly considered more subtle variation in relatedness beyond a simple paternity dichotomy (i.e., whether offspring were sired or not, e.g., Westneat and Sherman 1993; Kokko 1999; Houston and McNamara 2002; Sheldon 2002; Holen and Johnstone 2007; Kokko and Jennions 2008; Alonzo and Klug 2012). Furthermore, no empirical studies have quantified relatedness between socially‐paired males and the EPO they could rear, or quantified whether such relatedness arises primarily because cuckolded males are related to their socially‐paired female, or to their female's extra‐pair male, or both. Such metrics are required to consider the dynamics of paternal care that might arise given naturally occurring variation in male‐EPO relatedness, and to generate testable predictions explaining within‐ and among‐population variation.

Furthermore, it is rarely noted that in systems with variable biparental inbreeding, where individuals breed with differently related mates and are themselves inbred to different degrees due to inbreeding by their own parents, socially‐paired females and males can be differently related to within‐pair offspring (WPO) that they jointly produce and rear. Such variation in parent–offspring relatedness could potentially influence the optimal degrees of maternal versus paternal care for any brood, and thereby shape female–male cooperation or conflict over care, even without any extra‐pair reproduction (e.g., Parker et al. 2002; Houston et al. 2005). Such variation could further complicate the dynamics of joint parental care expressed within and across breeding attempts made by different individuals and pairs, and further complicate any change in a socially‐paired male's parental care that might be expected following female extra‐pair reproduction with related or unrelated extra‐pair males. However, no conceptual or empirical studies have explicitly quantified the degree to which females and their socially‐paired males are differently related to their jointly produced WPO. By the same logic, females can also be differently related to their own WPO versus EPO if they are differently related to their socially‐paired versus extra‐pair males, but no studies have explicitly quantified such variation. Consequently, no conceptual or empirical studies have quantified the overall relatedness of socially‐paired females and males to broods they could rear that comprise different numbers of EPO and WPO, or hence evaluated the opportunity for adaptive modulation of parental care given variable extra‐pair reproduction and biparental inbreeding.

Here, our two overall objectives are to provide a general conceptual framework that defines key metrics of parent–offspring relatedness in the context of extra‐pair reproduction and inbreeding, and to quantify variation in such relatedness arising in a natural system. First, we derive metrics that quantify the number of copies of any autosomal allele that is present in a socially‐paired female or male that is expected to be present identical‐by‐descent in WPO and EPO that these individuals produce or rear, as functions of the degrees to which individuals are themselves inbred and mate with related within‐pair and extra‐pair mates. We thereby define metrics that quantify the degree of allele‐sharing between WPO and EPO and their mother and her socially‐paired male given variable inbreeding.

Second, we use comprehensive pedigree data from free‐living song sparrows (*Melospiza melodia*) to quantify key metrics. Specifically, we quantify the degree to which socially‐paired females and males are expected to share identical‐by‐descent allele copies with (1) individual WPO that they jointly produce and rear; (2) with EPO that were sired by females’ extra‐pair males; and (3) with entire broods, comprising different numbers of WPO and EPO, for which the socially‐paired female and male could care. We explicitly quantify the reductions in allele‐sharing between a female's cuckolded socially‐paired male and individual EPO, and entire broods, compared to the WPO and broods the socially‐paired male could potentially have sired. We thereby quantify the allelic cost of a female's extra‐pair reproduction to her socially‐paired male. We then quantify the degree to which such reductions in allele‐sharing are ameliorated when extra‐pair and within‐pair reproduction occur among relatives. We thereby quantify the degree to which reproductive interactions among relatives can modulate the allelic cost of female extra‐pair reproduction to socially‐paired males. Finally, we discuss potential implications of such variation in parent–offspring relatedness for the modulation of paternal care following female extra‐pair reproduction, and for consequent coevolution of parental care, polyandry, and inbreeding.

## Derivation of Allelic Metrics

### RATIONALE

Basic expressions quantifying the expected degree of allele‐sharing between parents and offspring given inbreeding, and hence expected parent–offspring kinship, are long‐established in quantitative and population genetics (Falconer and Mackay 1996; Lynch and Walsh 1998). However, expressions pertaining specifically to WPO versus EPO have not been derived or parameterized in conceptual or empirical studies considering dynamics of extra‐pair reproduction and associated biparental care. Furthermore, long‐term changes in allele frequencies depend on the variance in allelic fitness as well as on mean fitness (Day and Otto 2001; Orr 2009). Most conclusively, alleles will be eliminated given zero fitness in any generation, meaning that geometric mean fitness is zero. Moreover, the combination of inbreeding and Mendelian sampling causes the realized degree of allele‐sharing between parents and offspring to vary around its expectation (e.g., Hill and Weir 2011, 2012; Kardos et al. 2015). Expressions that quantify the expected degree of allele‐sharing between parents and WPO and EPO, the probability that these offspring will carry more than zero copies of any parental allele, and the variance in allele‐sharing are consequently required to fully predict evolutionary dynamics. Accordingly, we derive metrics that quantify the expected allelic value, carrier probability, and allelic variance of WPO and EPO relative to their mother and her socially‐paired male, and highlight implications in the contexts of extra‐pair reproduction and parental care. Metrics derived for individual WPO and EPO can then be combined to quantify the total allelic values, carrier probabilities, and allelic variances of entire broods relative to their mother and her socially‐paired male, thereby quantifying the total allelic fitness that could result from parental care for any focal brood.

### WITHIN‐PAIR OFFSPRING

Consider a diploid system where focal individual *i* pairs with individual *j* and produces a WPO x (Appendix S1). The coefficient of kinship between i and j is *k*_ij_, which equals the probability that two homologous alleles sampled from i and j will be identical‐by‐descent (Lynch and Walsh 1998, p. 135). This probability is symmetrical (given diploidy), such that *k*_ij_ = *k*_ji_. Individual i's own coefficient of inbreeding is *f*_i_, which equals the probability that two homologous alleles within i are identical‐by‐descent (Lynch and Walsh 1998, p. 135). An individual's *f* therefore equals *k* between its genetic parents

The conditional probabilities that WPO x will carry zero, exactly one or two identical‐by‐descent copies of any autosomal allele that is present in focal parent i all depend on *f*_i_ and *k*_ij_ (Table 1A, Appendix S1). Consequently, the number of copies of any such allele that is expected to be present in x, hereafter the “allelic value” of x relative to i, is E(a_ix_) = 0.5 + 0.5*f*_i_ + *k*_ij_ (Table 1A, Appendix S1). The allelic value of any WPO x relative to parent i therefore increases with increasing *f*_i_ and *k*_ij_, and hence increases with the degree of inbreeding occurring in two generations. Furthermore, the probability that x will carry at least one identical‐by‐descent copy of an allele that is present in i (x's “carrier probability” relative to i, P(C_ix_)), and the expected number of allele copies in x per copy in i (E(a_ix_|a_i_)), also depend on *f*_i_ and *k*_ij_ (Table 1A, Appendix S1).

**Table 1:** Allelic metrics for (A) within‐pair offspring (WPO) and (B) extra‐pair offspring (EPO): the probabilities that WPO and EPO will carry zero, exactly one or two identical‐by‐descent copies of an autosomal allele that is present in a focal adult (P(a = 0), P(a = 1), and P(a = 2), respectively); “allelic value” E(a), the number of allele copies that is expected to be present in a WPO or EPO; “carrier probability” P(C), the probability that WPO and EPO will carry ≥1 allele copy; “allelic value per copy” E(a|a), the expected number of allele copies in a WPO and EPO per copy in the focal adult; and “allelic variance” var(a), the sampling variance in the number of allele copies in a WPO and EPO

Metric	(A) Within‐pair offspring	(B) Extra‐pair offspring		
Probability of zero copies	P(a_ix_ = 0)	0.5(1‐*f*_i_)(1‐*k*_ij_)	P(a_jy_ = 0)	1‐*k*_ji_‐*k*_jq_+*k*_ji_ *k*_jq_
Probability of one copy	P(a_ix_ = 1)	0.5+0.5*f*_i_‐*f*_i_ *k*_ij_	P(a_jy_ = 1)	*k*_ji_+*k*_jq_‐2*k*_ji_ *k*_jq_
Probability of two copies	P(a_ix_ = 2)	0.5*k*_ij_(1+*f*_i_)	P(a_jy_ = 2)	*k*_ji_ *k*_jq_
Allelic value	E(a_ix_)	0.5+0.5*f*_i_+*k*_ij_	E(a_jy_)	*k*_ji_+*k*_jq_
Carrier probability	P(C_ix_)	0.5(1+*f*_i_+*k*_ij_‐*f*_i_ *k*_ij_)	P(C_jy_)	*k*_ji_+*k*_jq_‐*k*_ji_ *k*_jq_
Allelic value per copy	E(a_ix_|a_i_)	(0.5+0.5*f*_i_+*k*_ij_)/(1+*f*_i_)	E(a_jy_|a_j_)	(*k*_ji_+*k*_jq_)/(1+*f*_j_)
Allelic variance	var(a_ix_)	0.25+*k*_ij_‐0.25*f*_i_^2^‐*k*_ij_^2^	var(a_jy_)	*k*_ji_+*k*_jq_‐*k*_ji_^2^‐*k*_jq_^2^

*f* is an adult's own coefficient of inbreeding. *k* is the coefficient of kinship between two adults. Subscripts refer to a focal female i, her socially‐paired male j, her extra‐pair mate q and her WPO x and EPO y. The allelic value E(a) of any offspring relative to its parent equals twice the parent–offspring coefficient of kinship. However the presented expressions explicitly reveal the relationships between E(a) and *f* and *k*, and thereby relate E(a) to current and previous inbreeding. Subtracting each metric for EPO from that for WPO gives the reduction in each metric due to extra‐pair reproduction. Derivations are summarized in Appendices S1 and S2.

In the expressions for WPO (Table 1A), the focal parent i could be the socially‐paired female or male (i.e., x's genetic mother or father). The coefficient of inbreeding *f*_j_ of the focal parent i's mate j (i.e., x's other parent) does not explicitly appear in these expressions (Table 1A). Any WPO x will therefore have different values of E(a_ix_), P(C_ix_), and E(a_ix_|a_i_) with respect to autosomal alleles carried by i and j if these parents are themselves inbred to different degrees (i.e., *f*_i_≠*f*_j_). If focal parent i is not inbred and does not inbreed (i.e., *f*_i_ = 0, *k*_ij_ = 0) then E(a_ix_), P(C_ix_), and E(a_ix_|a_i_) all reduce to the familiar parent–offspring value of 0.5. However, values can be much greater when x's parents and grandparents inbreed (i.e., *k*_ij_>0 and *f*_i_>0).

Because diploid offspring inherit alleles from diploid parents through Mendelian sampling, the realized number of identical‐by‐descent copies of any autosomal allele that is present in a focal parent i that is present in a WPO x will vary around the expectation E(a_ix_). The sampling variance var(a_ix_) itself depends on *f*_i_ and *k*_ij_, showing that inbreeding can systematically alter the distribution of allele‐sharing among parents and offspring (Table 1A, Appendix S1). Specifically, var(a_ix_) increases with *k*_ij_ but decreases with the squares of both *f*_i_ and *k*_ij_. Therefore, since 0 ≤ *f*_i_ ≤ 1 and 0 ≤ *k*_ij_ ≤ 1, var(a_ix_) increases with increasing *k*_ij_ but decreases slightly with increasing *f*_i_, and reduces to 0.25 if *f*_i_ = 0 and *k*_ij_ = 0.

### EXTRA‐PAIR OFFSPRING

Consider a focal male j that is paired with female i and is cuckolded by i's extra‐pair mate q, who sires i's EPO y (Appendix S2). The coefficients of kinship between j and i, and between j and q, are *k*_ji_ and *k*_jq_, respectively. The conditional probabilities that y will carry zero, exactly one or two identical‐by‐descent copies of any autosomal allele that is present in i's socially‐paired male j all depend on *k*_ji_ and *k*_jq_ (Table 1B, Appendix S2). The number of allele copies that is expected to be present in y, and hence y's allelic value with respect to j, is E(a_jy_) = *k*_ji_ + *k*_jq_ (Table 1B, Appendix S2). E(a_jy_) therefore increases linearly with increasing *k*_ji_ and *k*_jq_. The probability that y will carry at least one identical‐by‐descent copy of an allele that is present in j (y's “carrier probability” with respect to j, P(C_jy_)), the expected number of allele copies in y per copy in j (E(a_jy_|a_j_), and the sampling variance in the number of allele copies present in y (var(a_jy_)) also depend on *k*_ji_ and *k*_jq_ (Table 1B, Appendix S2). All metrics reduce to zero when male j is unrelated to his socially‐paired female i and her extra‐pair male q (i.e., *k*_ji_ = 0 and *k*_jq_ = 0).

## Empirical Methods

### STUDY SYSTEM AND PEDIGREE

Mandarte island, BC, Canada, holds a resident song sparrow population (recently 30 ± 12 SD breeding pairs per year) for which comprehensive long‐term paternity and pedigree data allow detailed analyses of the occurrence and consequences of extra‐pair reproduction and inbreeding (Reid et al. 2011, 2014a,b, 2015a,b,c; Lebigre et al. 2012). We used these data to calculate coefficients of inbreeding (*f*) and kinship (*k*) in and among breeding adults, and thereby calculate allelic metrics for WPO and EPO relative to their mother and her socially‐paired male (following Table 1).

Song sparrows are primarily socially monogamous; females and males form distinct breeding pairs and jointly defend territories and provision broods (Reid et al. 2015a,c). Pairs can rear up to three broods per year (Smith et al. 2006). However, there is frequent extra‐pair reproduction (Sardell et al. 2010; Reid et al. 2014b, 2015a). Males provision broods produced by their socially‐paired females, but do not provision broods produced by other females, even if they sired EPO in those broods.

Since 1975, all breeding attempts on Mandarte were monitored and chicks were color‐banded about 6 days after hatching (Smith et al. 2006). Mandarte lies within a large song sparrow metapopulation and receives approximately one immigrant per year on average, preventing inbreeding from escalating (Keller et al. 2001; Wolak and Reid 2016). Immigrants were color‐banded soon after arriving. The socially‐paired female and male that reared each brood of chicks were identified by their bands (Sardell et al. 2010; Reid et al. 2015b,c). These data were used to compile a pedigree linking banded chicks to their observed socially‐paired parents spanning 1975–2012 (Keller 1998; Reid et al. 2014b).

All banded chicks and adults alive during 1993–2012 were genotyped at 160 microsatellite loci (Nietlisbach et al. 2015). Each chick's true genetic parents were identified with >99% individual‐level statistical confidence, meaning that remaining parentage uncertainty is negligibly small (Reid et al. 2014b, 2015a). Overall, about 28% of chicks were assigned to extra‐pair males and hence identified as EPO (meaning that about 72% of chicks were WPO, see also Sardell et al. 2010). The pedigree was then corrected for extra‐pair paternity through 1993–2012 (Reid et al. 2014b, 2015a; Nietlisbach et al. 2015). Standard algorithms were used to calculate each individual's *f*, and *k* between pairs of individuals, directly from the full 1975–2012 pedigree (i.e., relative to the 1975 baseline, Keller 1998; Reid et al. 2014b, 2015a). These calculations provide expected values of *f* and *k* given pedigree relationships, and do not directly utilize microsatellite genotypes.

### INDIVIDUAL OFFSPRING ANALYSES

For each WPO, values of *f*_i_ and *k*_ij_ were extracted taking each chick's mother and then father (i.e., the mother's socially‐paired male) as focal parent i. The allelic metrics E(a_ix_), P(C_ix_), E(a_ix_|a_i_), and var(a_ix_) were calculated for each WPO relative to each parent (Table 1A). For each EPO, values of *k*_ji_ and *k*_jq_ were extracted. The allelic metrics E(a_jy_), P(C_jy_), E(a_jy_|a_j_), and var(a_jy_) were calculated for each EPO relative to its mother i's socially‐paired male j (Table 1B). Allelic metrics for each EPO relative to its mother were calculated by defining the mother and her extra‐pair male (i.e., the EPO's sire) as individuals i and j and parameterizing the expressions for WPO (Table 1A). The distributions of these metrics were summarized across all observed WPO and EPO. To quantify whether females or their socially‐paired males were systematically more or less closely related to their jointly produced WPO, the distribution of the difference in each WPO's allelic metrics relative to its mother versus father was computed.

To quantify the cost of female extra‐pair reproduction to a female's socially‐paired male in terms of the decrease in identical‐by‐descent allele copies present in each EPO a male reared, allelic metrics for each observed EPO relative to its mother's socially‐paired male were also calculated as if the male had sired the EPO (i.e., produced a WPO). Similarly, to quantify the allelic cost or benefit of extra‐pair reproduction to a female, allelic metrics for each potential WPO were also calculated relative to their mother. Metrics for hypothetical WPO can be calculated because required values of *f*_i_ and *k*_ij_ for socially‐paired females and males can be computed from the pedigree, even if no real WPO were produced. The distributions of the differences in allelic metrics between socially‐paired females’ and males’ potential WPO and observed EPO were computed. Linear regressions were fitted to quantify the degree to which the differences in allelic metrics between observed or potential WPO and observed EPO relative to their mother and her socially‐paired male was explained by realized variation in *k*_ij_ and *k*_jq_.

### BROOD ANALYSES

To quantify the total allelic value of each observed brood relative to its mother versus her socially‐paired male, the allelic values of each chick calculated relative to each socially‐paired parent were summed across all chicks in each brood. The probability that each brood would carry at least one identical‐by‐descent copy of any autosomal allele that is present in each socially‐paired parent (i.e., the total brood carrier probability, representing the probability that brood‐level parental care could facilitate persistence of an allele that is present in the focal parent) was calculated as one minus the product of the probabilities that each chick would carry zero allele copies. The variance in the total number of allele copies expected to be present in any brood was directly calculated as the difference between the expectation of squared allelic value and the squared expectation of allelic value.

To quantify the total cost of female extra‐pair reproduction to a female's socially‐paired male in terms of the decrease in identical‐by‐descent allele copies present in each brood a male reared, allelic metrics for each brood were also calculated as if the socially‐paired male had sired all chicks (i.e., all WPO). To quantify the degree to which this cost of extra‐pair reproduction was decreased by reproduction among relatives, allelic metrics for each observed brood relative to its mother's socially‐paired male, and for the male's potential brood of WPO, were also calculated as if there had been no inbreeding or pairing among relatives (i.e., specifying *f*_i_ = 0, *k*_ji_ = 0, and *k*_jq_ = 0). The distributions of the absolute and proportional differences between the allelic metrics for the potential and observed broods were computed.

### DATA RESTRICTIONS

Analyses were restricted to broods where ≥1 offspring survived to banding and paternity assignment, and where the socially‐paired female and male and any extra‐pair sires had all hatched since 1993 or were immigrants. Immigrants were defined as unrelated to existing population members at arrival (i.e., *k* = 0, Reid et al. 2006; Wolak and Reid 2016). All focal parents therefore had genetically verified parents, or were defined as unrelated to their mates, thereby eliminating pedigree error up to focal offsprings’ grandparents (Reid et al. 2015a). Although there will still be some pedigree error concerning misassigned great‐grandfathers and more distant ancestors, most focal offspring had ≥3 generations of genetically verified ancestors, and the impact of pedigree error concerning more distant ancestors on estimates of *f* and *k* among contemporary individuals is very small (Reid et al. 2015a). For current analyses, nine immigrant females and four immigrant males that contributed observed offspring were assumed to be outbred (i.e., *f* = 0), but overall conclusions were similar if they were assumed to be somewhat inbred (e.g., *f* = 0.05), or if their offspring were excluded from analyses. For reference, values of *k* = 0, 0.0625, 0.125, and 0.25 equate to pairings between unrelated individuals and outbred third‐order, second‐order, and first‐order relatives, respectively. Equivalent values of *f* equate to offspring of such pairings.

As for any pedigree analysis, calculations assume weak selection on any allele (Charlesworth and Charlesworth 1987). Furthermore, pedigree analyses provide expected values of *k* and *f*, which can differ from realized values following Mendelian sampling (Hill and Weir 2011; Kardos et al. 2015). Realized *k* and *f* can potentially be accurately estimated from high‐density genetic marker data. However, realized *k* between males and females and hypothetical WPO that they did not produce cannot, by definition, be measured. Consequently, the difference in realized allelic value between individuals’ observed EPO and potential WPO cannot be measured, and cannot be known a priori by individuals enacting extra‐pair reproduction. Furthermore, the realized allelic values of broods containing multiple offspring will converge toward expectation (see *Discussion*). Pedigree analysis is therefore an appropriate framework with which to conceptualize and quantify variation in allelic metrics for offspring and broods relative to their various parents following inbreeding and extra‐pair reproduction.

Analyses were run in R (version 3.2.2, R Core Team 2013) using package kinship2. Raw means are presented ±1 SD. Regression slopes are presented ±1 standard error (SE). Data are available from the Dryad Digital Repository: http://dx.doi.org/10.5061/dryad.4r383.

## Results

The dataset comprised 741 broods hatched during 1994–2012, totaling 2087 offspring that comprised 1526 (73.1%) WPO and 561 (26.9%) EPO.

### WPO DATA

The 1526 observed WPO occurred in 639 broods produced by 196 females and 200 socially‐paired males (that sired and reared the WPO), spanning 318 different social pairings. Across the individual parents, mean female *f* was 0.062 ± 0.042 (range 0.000–0.200, 12.7% zeros) and mean male *f* was 0.064 ± 0.038 (range 0.000–0.200, 12.5% zeros). Across the 318 pairings, mean *k*_ij_ was 0.084 ± 0.061 (range 0.000–0.356, 8.2% zeros). Female *f* and male *f* were both positively correlated with *k*_ij_ (Pearson's correlation coefficients: *r*_p_ = 0.29 and 0.33, respectively, as previously observed in the study population, Reid et al. 2006).

### WPO VALUES TO MOTHER AND FATHER

**Figure 1:**
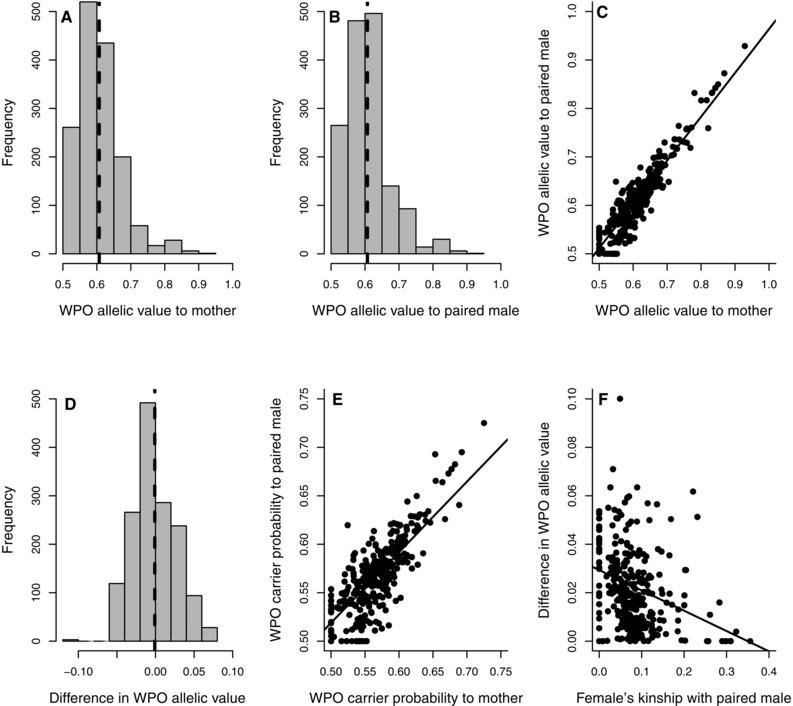
Variation in allelic metrics across 1526 observed within‐pair offspring (WPO): (A and B) the distributions of WPOs’ allelic values E(a_ix_) relative to their (A) mother and (B) father (i.e., the mother's socially‐paired male); (C) the relationship between WPOs’ E(a_ix_) values relative to their mother versus father; (D) the distribution of the difference in WPOs’ E(a_ix_) values relative to their mother versus father; (E) the relationship between WPOs’ carrier probabilities P(C_ix_) relative to their mother versus father; and (F) the relationship between a female's kinship with her socially‐paired male (*k*_ij_) and the absolute difference in WPOs’ E(a_ix_) values relative to their mother versus father. Dashed lines depict means, solid lines depict linear regressions.

Because *f*_i_ and/or *k*_ij_ commonly exceeded zero, allelic metrics for WPO relative to their socially‐paired mother and father varied substantially across the 1526 observed WPO (Table 2A). Mean allelic value E(a_ix_) was 0.607 ± 0.066 relative to both parents (Table 2A, Fig. 1A and B), and E(a_ix_) exceeded the value of 0.5 expected given no inbreeding in 1433 (93.9%) and 1484 (97.2%) WPO relative to their mother and father, respectively. The carrier probabilities P(C_ix_), allelic values per copy in i E(a_ix_|a_i_) and allelic variances var(a_ix_) showed similar variation (Table 2A, Fig. S1).

**Table 2:** Mean ± 1 SD (and range) of allelic value E(a), carrier probability P(C), allelic value per copy in a focal adult E(a|a), and allelic variance var(a) across (A) 1526 observed within‐pair offspring (WPO), (B) 561 observed extra‐pair offspring (EPO), (C) 561 potential WPO that a female's socially‐paired male could have sired and (D) the difference between 561 potential WPO and observed EPO relative to their mother and her socially‐paired male, and (E) the difference in each metric between 561 observed EPO relative to their mother versus her socially‐paired male

	Offspring	Parent	E(a)	P(C)	E(a|a)	var(a)
(A)	WPO	Male	0.607 ± 0.066	0.565 ± 0.037	0.574 ± 0.053	0.318 ± 0.043
			(0.500–0.929)	(0.500–0.725)	(0.500–0.810)	(0.247–0.474)
		Mother	0.607 ± 0.066	0.565 ± 0.037	0.574 ± 0.053	0.318 ± 0.043
			(0.500–0.929)	(0.500–0.725)	(0.500–0.810)	(0.247–0.474)
(B)	EPO	Male	0.172 ± 0.085	0.164 ± 0.078	0.161 ± 0.078	0.150 ± 0.064
			(0.000–0.470)	(0.000–0.430)	(0.000–0.410)	(0.000–0.331)
		Mother	0.612 ± 0.060	0.569 ± 0.035	0.575 ± 0.047	0.320 ± 0.039
			(0.500–0.842)	(0.500–0.678)	(0.500–0.783)	(0.247–0.460)
(C)	Potential WPO	Male	0.622 ± 0.072	0.573 ± 0.040	0.585 ± 0.059	0.327 ± 0.046
			(0.500–0.929)	(0.500–0.725)	(0.500–0.810)	(0.248–0.474)
		Mother	0.622 ± 0.074	0.573 ± 0.041	0.585 ± 0.058	0.327 ± 0.046
			(0.500–0.929)	(0.500–0.725)	(0.500–0.810)	(0.248–0.474)
(D)	Potential WPO minus EPO	Male	0.450 ± 0.049	0.409 ± 0.053	0.424 ± 0.048	0.177 ± 0.039
			(0.221–0.564)	(0.198–0.507)	(0.202–0.500)	(0.028–0.250)
		Mother	0.010 ± 0.076	0.005 ± 0.035	0.009 ± 0.071	0.007 ± 0.055
			(−0.269–0.245)	(−0.124–0.114)	(−0.250–0.230)	(−0.197–0.159)
(E)	Difference in		0.440 ± 0.087	0.405 ± 0.074	0.415 ± 0.082	0.170 ± 0.065
	EPO value to mother versus male		(0.179–0.689)	(0.176–0.591)	(0.170–0.693)	(−0.001–0.375)

Values of E(a_ix_) relative to each WPO's mother and father were strongly positively, but not perfectly, correlated across WPO (*r*_p_ = 0.91, Fig. 1C). The difference from *r*_p_ = 1 arose because *f* differed between a WPO's mother and father for 1470 (96.3%) of 1526 WPO (mean absolute difference 0.045 ± 0.034). The mean difference in E(a_ix_) relative to a WPO's mother versus father was approximately zero (−0.001 ± 0.028, Fig. 1D). Therefore, WPO were not systematically more or less closely related to their mother than to their father. However, the mean absolute difference in a WPO's E(a_ix_) relative to its two parents was 0.023 ± 0.017 (maximum 0.100). Furthermore, WPOs’ P(C_ix_) values relative to their mother versus father were also positively, but not perfectly, correlated (*r*_p_ = 0.74, Fig. 1E), and the mean absolute difference was 0.021 ± 0.016. There were therefore commonly asymmetries in expected allelic value and carrier probability between socially‐paired females and males and the WPO that they jointly produced and reared. However, E(a_ix_|a_i_) and var(a_ix_) were tightly correlated across a WPO's two parents (*r*_p_ ≈ 0.99, Fig. S2), and hence differed little between the two parents.

As expected, the absolute difference in a WPO's E(a_ix_) relative to its mother versus father decreased with increasing *k*_ij_ (*r*_p_ = −0.28, linear regression slope: β_1524_ = −0.08 ± 0.01, Fig. 1F), as did the absolute difference in P(C_ix_) (*r*_p_ = −0.34, β_1524_ = −0.10 ± 0.01). WPO therefore had more similar allelic values and carrier probabilities relative to their mother and father when these parents were more closely related.

### EPO DATA

The 561 observed EPO occurred in 321 broods, and were produced by 133 females and 121 extra‐pair sires and reared by 144 socially‐paired males. These individuals formed 198 different social pairings, 254 female extra‐pair male pairs and 258 sets of associated socially‐paired and extra‐pair males. Across the parents, mean female *f* was 0.064 ± 0.043 (range 0.000–0.200, 15.8% zeros), mean extra‐pair male *f* was 0.058 ± 0.036 (range 0.000–0.200, 11.6% zeros), and mean socially‐paired male *f* was 0.063 ± 0.041 (range 0.000–0.257, 9.7% zeros). Mean *k*_ji_ between a socially‐paired male and female was 0.090 ± 0.062 (range 0.000–0.356, 6.6% zeros), and mean *k*_jq_ between a socially‐paired male and his female's extra‐pair male was 0.083 ± 0.053 (range 0.000–0.325, 4.7% zeros). *k*_ji_ and *k*_jq_ were not strongly correlated across EPO (*r*_p_ = 0.09).

### EPO VALUES TO SOCIALLY‐PAIRED MALE

**Figure 2:**
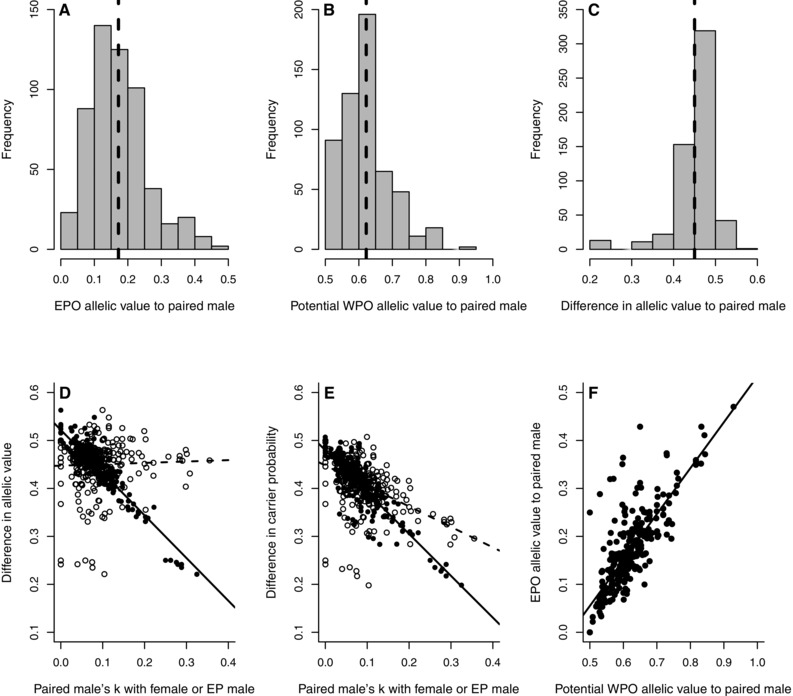
(A–C) Distributions of (A) allelic value E(a_jy_) of 561 observed extra‐pair offspring (EPO) relative to their mother's socially‐paired male, (B) allelic value E(a_jx_) of 561 potential within‐pair offspring (WPO) that these males could have sired, and (C) the difference in allelic value between the male's potential WPO and observed EPO. (D–F) Relationships between (D) the difference in allelic value and (E) the difference in carrier probability and the male's kinship (*k*) with his socially‐paired female (open symbols) and her extra‐pair male (EP male, filled symbols), and (F) the allelic values of the male's potential WPO versus observed EPO. Dashed lines depict means, solid lines depict linear regressions. Note that x‐axis scales differ between A and B, and that x‐ and y‐axis scales differ on F.

Because *k*_ji_ and/or *k*_jq_ commonly exceeded zero, allelic metrics of EPO relative to their mother's socially‐paired male varied substantially across the 561 observed EPO (Table 2B). Specifically, mean allelic value E(a_jy_) was 0.172 ± 0.085, and E(a_jy_) equaled zero for only 14 (2.5%) EPO (Fig. 2A). Cuckolded socially‐paired males were therefore almost always detectably related to EPO that they reared. Indeed, the value of an EPO to its mother's socially‐paired male was occasionally almost as high as the value of a WPO (maximum E(a_jy_) of 0.470 vs. minimum E(a_jx_) of 0.500, Table 2A and B). The carrier probability P(C_jy_) of an EPO relative to its mother's socially‐paired male, the allelic value per copy in the male E(a_jy_|a_j_), and the allelic variance var(a_jy_) showed similar variation (Table 2B, Fig. S3).

### DECREASE IN OFFSPRING VALUE TO SOCIALLY‐PAIRED MALE

If females’ socially‐paired males had sired the EPO they reared (i.e., produced WPO), the allelic metrics of those potential WPO would have been similar to those for males’ observed WPO (Table 2A and C). For example, mean E(a_jy_) would have been 0.622 ± 0.072 (Fig. 2B). The mean allelic value of an EPO to a socially‐paired male of 0.172 was therefore about 28% of that of the male's potential WPO (Table 2B and C). More precisely, the mean difference in allelic value between a socially‐paired male's potential WPO and observed EPO was 0.450 ± 0.049 (Table 2D, Fig. 2C). The differences in carrier probability, allelic value per copy, and allelic variance showed similar variation (Table 2D, Fig. S3).

As expected, the difference in allelic value between a socially‐paired male's potential WPO and observed EPO did not vary with *k*_ji_, but decreased with increasing *k*_jq_ and increased slightly with increasing *f*_j_ (Table 3, Figs. 2D and S4). Meanwhile, the difference in carrier probability decreased with increasing *k*_ji_ and *k*_jq_ and decreased weakly with increasing *f*_j_ (Table 3, Figs. 2E and S4). The differences in allelic value per copy and allelic variance decreased with increasing *k*_jq_ and *f*_j_, but did not vary with *k*_ji_ (Table 3, Fig. S4). These relationships illustrate that the cost of female extra‐pair reproduction to a socially‐paired male, measured as decreases in allelic metrics of individual EPO, was smaller when the male was related to his socially‐paired female and to her extra‐pair male. Furthermore, allelic metrics for socially‐paired males’ EPO were positively correlated with metrics for these males’ potential WPO, and hence with any WPO that a male sired in the same brood (*r*_p_ ≈ 0.8 for all four metrics, Figs. 2F and S5).

**Table 3:** Slopes (±1 standard error) of linear regressions of the difference in allelic value, carrier probability, allelic value per copy, and allelic variance between a socially‐paired male's potential within‐pair offspring (WPO) and observed extra‐pair offspring (EPO) and his coefficients of kinship with his (A) socially‐paired female (*k*_ji_) and (B) his socially‐paired female's extra‐pair male (*k*_jq_), and (C) his own coefficient of inbreeding (*f*_j_) across 561 EPO

	(A) *k*_ji_	(B) *k*_jq_	(C) *f*_j_
Allelic value	0.03 ± 0.03	−0.89 ± 0.02	0.14 ± 0.05
Carrier probability	−0.43 ± 0.03	−0.87 ± 0.02	−0.12 ± 0.06
Allelic value per copy	−0.06 ± 0.03	−0.93 ± 0.01	−0.28 ± 0.05
Allelic variance	−0.08 ± 0.03	−0.76 ± 0.01	−0.36 ± 0.04

### EPO VALUES TO MOTHER

**Figure 3:**
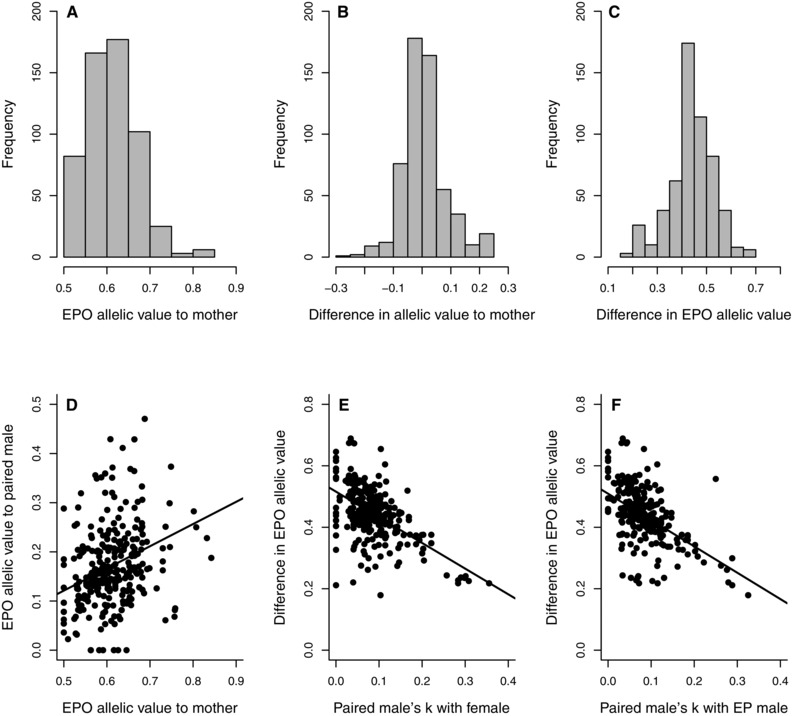
(A–C) Distributions of (A) allelic value E(a_iy_) of 561 observed extra‐pair offspring (EPO) relative to their mothers, (B) the difference in allelic value between a female's observed EPO and her potential within‐pair offspring (WPO) and (C) the difference in allelic value of observed EPO relative to their mother versus her socially‐paired male. (D) Relationship between an EPO's allelic value relative to its mother versus her socially‐paired male. (E and F) Relationships between the difference in an EPO's allelic value relative to its mother versus her socially‐paired male and (E) the kinship *k*_ij_ between these individuals, and (F) the kinship *k*_jq_ between the female's socially‐paired male and extra‐pair male (EP male). Lines depict linear regressions.

Allelic metrics for EPO relative to their mother varied substantially across the 561 observed EPO (Table 2B). For example, mean E(a_iy_) was 0.612 ± 0.060, and E(a_iy_) exceeded 0.5 for 537 (95.7%) EPO (Table 2B, Fig. 3A). The distributions of metrics across observed EPO were similar to the distribution across the females’ 1526 observed WPO (Table 2A), and across the 561 potential WPO a female could have produced with her socially‐paired male (Table 2C). Consequently, the difference in each allelic metric between a female's potential WPO and her observed EPO also varied substantially, but the means were close to zero (Table 2D, Figs. 3B and S6). Therefore, on average, females did not directionally adjust the allelic metrics of their offspring through extra‐pair reproduction.

### EPO VALUES TO MOTHER VERSUS SOCIALLY‐PAIRED MALE

The allelic metrics of an EPO relative to its mother of course always substantially exceeded its metrics relative to its mother's socially‐paired male (Table 2E, Figs. 3C and S7); for example, the mean difference in allelic value was 0.440 ± 0.087. However, allelic metrics calculated relative to an EPO's mother were moderately positively correlated with metrics calculated relative to the mother's socially paired male across the 561 observed EPO (Table 4A, Figs. 3D and S8). The difference in each metric relative to an EPO's mother versus her socially‐paired male decreased with increasing *k*_ij_ and *k*_jq_ (Table 4B,C, Figs. 3E and F and S8). Therefore, as expected, the difference in allelic metrics of an EPO relative to a female versus her socially‐paired male decreased when these paired individuals were more closely related, and when the socially‐paired male was more closely related to the female's extra‐pair male.

**Table 4:** (A) Pearson's correlation coefficients (*r*_p_) between four allelic metrics calculated relative to an extra‐pair offspring's mother versus her socially‐paired male, and Pearson's correlation coefficients and linear regression slopes (β ± 1 standard error) between the difference in each metric relative to an extra‐pair offspring's mother versus her socially‐paired male and the coefficient of kinship between (B) the socially‐paired female and male (*k*_ji_), and (C) the female's socially‐paired and extra‐pair males (*k*_jq_), across 561 observed extra‐pair offspring

		(B) *k*_ji_		(C) *k*_jq_	
	(A)				
	*r*_p_	*r*_p_	β	*r*_p_	β
Allelic value	0.32	−0.61	−0.84 ± 0.05	−0.50	−0.86 ± 0.06
Carrier probability	0.34	−0.70	−0.82 ± 0.04	−0.62	−0.90 ± 0.05
Allelic value per copy	0.21	−0.69	−0.90 ± 0.04	−0.51	−0.82 ± 0.06
Allelic variance	0.28	−0.69	−0.71 ± 0.03	−0.52	−0.67 ± 0.05

### BROOD DATA

The 741 observed broods were reared by 203 females and 212 socially‐paired males, spanning 342 different social pairings. Mean brood size was 2.8 ± 0.9 chicks (median 3, range 1–4). Overall, 321 broods (43.3%) contained at least one observed EPO (hereafter “EPO‐broods”), and 420 broods (56.7%) contained only WPO (hereafter “WPO‐broods”).

**Figure 4:**
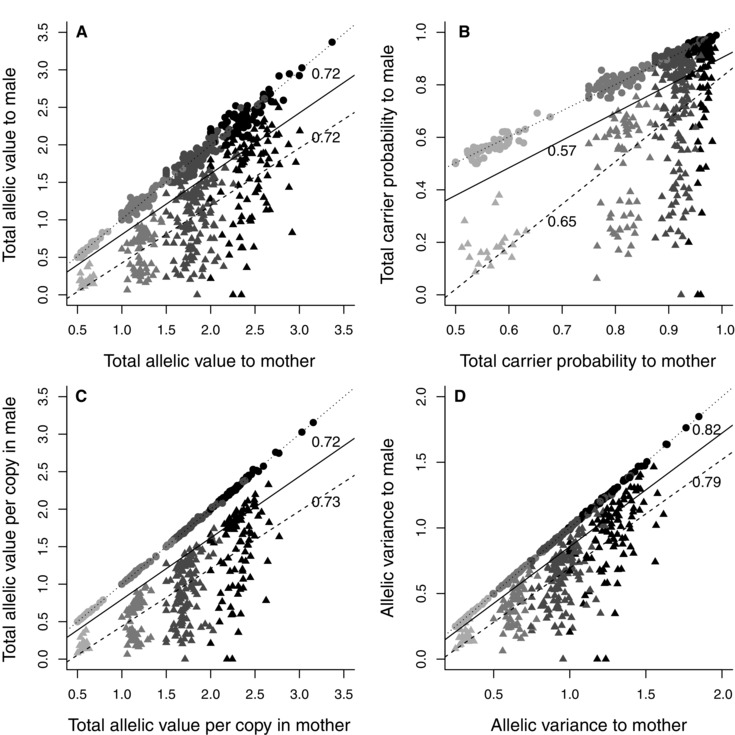
Relationships between the total (A) allelic value, (B) carrier probability, (C) allelic value per copy in each focal adult, and (D) allelic variance of an observed brood relative to its mother versus her socially‐paired male. Points and triangles denote observed WPO‐broods (*N* = 420) and EPO‐broods (*N* = 321), respectively. Black, dark‐gray, mid‐gray, and light‐gray symbols denote brood sizes of four, three, two, and one chick, respectively. Solid, dotted, and dashed lines depict linear regressions fitted through all observed broods, WPO‐broods and EPO‐broods, respectively. Pearson's correlation coefficients calculated across all broods and EPO‐broods are adjacent to regression lines. Correlations were 0.99 across WPO‐broods.

Across all 741 broods, the brood's total allelic value, carrier probability, allelic value per copy, and allelic variance calculated relative to its mother were all strongly positively correlated with brood size (0.91 ≤ *r*_p_ ≤ 0.97, Figs. 4 and S9). However variation in *f*_i_ and *k*_ij_ was sufficient to cause values to overlap across brood sizes; some broods of two and three chicks had higher values of allelic metrics than some broods of three and four chicks, respectively (Figs. 4 and S9). The positive correlations between brood size and brood allelic metrics calculated relative to the mother's socially‐paired male were weaker because some broods contained EPO (0.55 ≤ *r*_p_ ≤ 0.76), and values overlapped substantially across all brood sizes (Figs. 4 and S9).

### BROOD VALUE TO MOTHER VERSUS SOCIALLY‐PAIRED MALE

Across all 741 broods, a brood's total allelic value, carrier probability, allelic value per copy, and allelic variance calculated relative to its mother were all positively correlated with those calculated relative to the mother's socially‐paired male (Fig. 4). These correlations were very strong across the 420 WPO‐broods (Fig. 4). However, metrics for WPO‐broods calculated relative to their mother versus father typically differed to some degree (Table 5A, Fig. S10). For example, the mean absolute difference in total brood allelic value was 0.062 ± 0.057 (Table 5A, Fig. S10), equating to a mean of 3.7 ± 3.0% of the brood's value to each parent. The mean absolute differences in the other three allelic metrics were smaller (Table 5A, Fig. S10).

**Figure 5:**
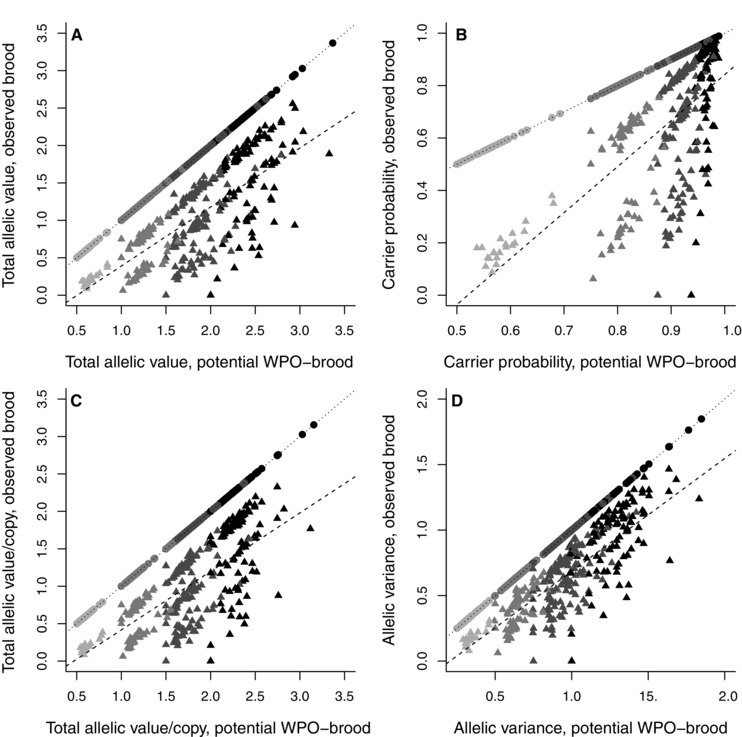
Relationships between the total (A) allelic value, (B) carrier probability, (C) allelic value per copy in the focal male, and (D) allelic variance of an observed brood relative to its mother's socially‐paired male, versus the paired male's potential WPO‐brood. Points and triangles denote observed WPO‐broods (*N* = 420) and EPO‐broods (*N* = 321), respectively. Black, dark‐gray, mid‐gray, and light‐gray symbols denote brood sizes of four, three, two, and one chick, respectively. Dotted and dashed lines depict linear regressions fitted through observed WPO‐broods and EPO‐broods, respectively.

**Table 5:** Mean ± 1 SD (and range) of the difference in total brood allelic value E(a), carrier probability P(C), allelic value per copy E(a|a), and allelic variance var(a) across (A) 420 broods that contained only within‐pair offspring (WPO‐broods) relative to their mother versus father; (B) 321 broods that contained at least one extra‐pair offspring (EPO‐broods) relative to their mother versus her socially‐paired male; (C) 321 potential WPO‐broods versus observed EPO‐broods relative to the mother's socially‐paired male; and (D) 321 potential WPO‐broods versus observed EPO‐broods relative to the socially‐paired male given no inbreeding

	Comparison	E(a)	P(C)	E(a|a)	var(a)
(A)	WPO‐brood to mother versus father	0.062 ± 0.057	0.013 ± 0.012	0.007 ± 0.008	0.004 ± 0.004
		(0.000–0.284)	(0.000–0.081)	(0.000–0.054)	(0.000–0.023)
(B)	EPO‐brood to mother versus paired male	0.765 ± 0.435	0.239 ± 0.203	0.726 ± 0.401	0.297 ± 0.199
		(0.152–2.347)	(0.006–0.960)	(0.211–2.244)	(−0.003–1.237)
(C)	Potential WPO‐brood versus observed	0.787 ± 0.413	0.242 ± 0.201	0.742 ± 0.392	0.310 ± 0.179
	EPO‐brood to paired male	(0.242–2.009)	(0.014–0.938)	(0.228–2.000)	(0.044‐1.000)
		*0.46* ± *0.22*	*0.29* ± *0.25*	*0.46* ± *0.22*	*0.34* ± *0.19*
		*(0.12–1.00)*	*(0.01–1.00)*	*(0.12–1.00)*	*(0.05–1.00)*
(D)	Potential WPO‐brood versus observed	0.874 ± 0.442	0.874 ± 0.442	0.874 ± 0.442	0.437 ± 0.221
	EPO‐brood to paired male given no	(0.500–2.000)	(0.500–2.000)	(0.500–2.000)	(0.250–1.000)
	inbreeding	*0.63* ± *0.29*	*0.63* ± *0.29*	*0.63* ± *0.29*	*0.63* ± *0.29*
		*(0.25–1.00)*	*(0.25–1.00)*	*(0.25–1.00)*	*(0.25–1.00)*

In (C and D) the mean ± 1 SD (and range) proportional differences between a socially‐paired male's potential WPO‐brood and observed EPO‐brood (relative to the potential WPO‐brood) are shown in italics.

The positive correlations between brood allelic metrics calculated relative to a brood's mother versus her socially‐paired male were weaker across the 321 EPO‐broods (Fig. 4). Due to female extra‐pair reproduction, values for the female's socially‐paired male were always lower than those for the female (Table 5B, Fig. S11). For example, the mean difference in total allelic value of an observed EPO‐brood relative to its mother versus her socially‐paired male was 0.765±0.435, which is equivalent to the value of about 1.5 outbred offspring (Table 5B, Fig. S11).

### DECREASE IN BROOD VALUE TO SOCIALLY‐PAIRED MALE

Socially‐paired males that were cuckolded to some degree, and hence reared an EPO‐brood, always experienced a decrease in all four brood allelic metrics compared to the WPO‐brood they would have produced had they sired all their paired female's offspring (Table 5C, Figs. 5 and S12). The proportional decreases were substantial, for example equating to means of 46 and 29% of total brood allelic value and carrier probability, respectively (Table 5C, Fig. S13).

If *f*_j_, *k*_ji_, and *k*_jq_ had all been zero (i.e., no inbreeding by the previous or current generation), the mean absolute decrease in total brood allelic value, carrier probability, and allelic value per copy between a male's potential WPO‐brood and observed EPO‐brood would all have been 0.874 ± 0.442 (Table 5D, Fig. S14). The mean proportional decreases would have been 0.63 ± 0.29 (Table 5D, Fig. S14). The absolute and proportional decreases in these metrics that males experienced due to cuckoldry were therefore ameliorated by about 10% and 27% by the occurrence of social pairing and extra‐pair reproduction among relatives. The mean absolute and proportional decreases in allelic variance would have been 0.437 ± 0.221 and 0.63 ± 0.29, respectively, given no inbreeding (Table 5D, Fig. S14), and were therefore reduced by 29% and 46% by the occurrence of reproduction among relatives.

## Discussion

It is widely hypothesized that, under some circumstances, cuckolded males should reduce paternal care for broods that contain EPO, reducing the fitness of their socially‐paired female's entire brood of offspring and causing selection against female extra‐pair reproduction (Westneat and Sherman 1993; Sheldon 2002; Arnqvist and Kirkpatrick 2005; Kokko and Jennions 2008; Alonzo and Klug 2012). However, no empirical studies have quantified the degree to which males are in fact related to their socially‐paired female's EPO that they did not sire, or quantified the degree to which socially‐paired females and males are differently related to their jointly produced WPO. Consequently, no studies have quantified total allelic values of broods containing different numbers of EPO and/or WPO relative to their mother and her socially‐paired male. There is, therefore, no empirical basis on which the consequences of emerging variation for the dynamics of parental care, or hence for resulting selection against female extra‐pair reproduction, can be examined. We show that socially‐paired male song sparrows are commonly related to EPO that they reared but did not sire, and that socially‐paired females and males are commonly differently related to their jointly produced WPO, creating complex patterns of kinship within nuclear families that could potentially influence the coevolution of parental care, extra‐pair reproduction, and inbreeding.

### WITHIN‐PAIR OFFSPRING

Theory shows that parent–offspring kinship in diploid systems, and the corresponding expected allelic values of offspring to their parents, are no longer uniformly 0.25 and 0.5, respectively, when inbreeding occurs in the current or previous generation (Table 1A, Lynch and Walsh 1998). However, variation in parent–offspring kinship is surprisingly rarely mentioned or explicitly quantified by empiricists studying variation in parental care in vertebrates, even when inbreeding occurs (but see Margulis 1997). In song sparrows, offspring allelic values and carrier probabilities almost always exceeded 0.5, and increments per WPO were sometimes sufficient to cause total brood values to overlap across brood sizes. Because increments are additive across offspring, such overlaps could be even greater in species with larger brood sizes. Consequently, in systems where variable inbreeding occurs, the absolute and relative allelic values of broods to any (potentially) caring parent cannot be accurately quantified simply by counting the number of offspring present, even with no extra‐pair reproduction.

Furthermore, socially‐paired females and males were often differently related to WPO that they jointly produced and reared. The total allelic values and carrier probabilities of WPO‐broods consequently differed between the brood's two genetic parents. These differences arose when socially‐paired females and males were themselves inbred to different degrees, reflecting different degrees of inbreeding by WPOs’ maternal versus paternal grandparents. Differences in the optimal degrees of paternal and maternal care for jointly reared WPO, and consequent conflict over such parental care (e.g., Parker et al. 2002; Houston et al. 2005), might potentially be exacerbated or ameliorated by such asymmetries. Furthermore, WPO had more similar allelic metrics relative to their mother and father when these parents were more closely related, implying that social pairing between relatives might further modulate conflict over parental care (see also Thünken et al. 2007). In song sparrows, the absolute differences in allelic metrics for WPO‐broods relative to their mother versus father were small. Parental care strategies in such systems therefore seem unlikely to be overwhelmingly influenced by asymmetrical kinship between WPO and their two parents. However, our conceptual framework highlights that such asymmetries can arise, and would be greater in systems where differences in *f* between paired parents are greater. This could arise when close inbreeding (e.g., sib‐mating) and outbreeding co‐occur, and hence when highly inbred and outbred individuals commonly mate.

### EXTRA‐PAIR OFFSPRING

The allelic values and carrier probabilities of EPO relative to their mother's socially‐paired male were always lower than the values of the corresponding WPO that the male could potentially have sired. However, because male song sparrows were commonly related to their socially‐paired female and/or to her extra‐pair male, allelic metrics for EPO almost always exceeded zero and sometimes approached values observed for WPO. Consequently, although extra‐pair reproduction always decreased the value of a female's brood to her cuckolded socially‐paired male, the magnitude of the decrease was reduced by social pairing and extra‐pair reproduction among relatives. Such reproductive interactions among relatives therefore reduced the probability that socially‐paired males could invest costly paternal care in broods that contained zero or few identical‐by‐descent copies of their own alleles. Reproduction among relatives might therefore increase the evolutionary benefit of paternal care following cuckoldry, thereby reducing selection on cuckolded males to decrease paternal care, and reducing consequent selection against female extra‐pair reproduction.

Mendelian sampling generates variance in the realized number of identical‐by‐descent copies of any parental allele that is present in any individual offspring (Hill and Weir 2011). The song sparrow data illustrate that this variance can substantially exceed the basic value of 0.25 (for a single locus) when inbreeding occurs, creating substantial variance in the number of parental allele copies present in entire broods. Meanwhile, extra‐pair reproduction decreased a brood's total allelic variance relative to a male versus his socially‐paired female, and decreased the total allelic variance of a male's observed EPO‐brood compared to the WPO‐brood he could potentially have produced. Cuckoldry therefore decreased the variance in the number of copies of any allele that is present in a socially‐paired male that will be present in a brood for which the male could care. However this decrease reflects a strong positive mean‐variance relationship in allelic value, which arises because both quantities depend on brood size, *k*_ij_ and *k*_jq_ (Table 1, Fig. S15). Extra‐pair reproduction, and consequent cuckoldry, therefore does not readily allow males to reduce a brood's allelic variance independently of its expected allelic value.

Furthermore, the variance in total genome‐wide kinship between a socially‐paired male and his entire dependent brood will depend on the total number of independently inherited genome segments. This will in turn depend on chromosome number and recombination rate (Hill and Weir 2011, 2012) and on brood size and could consequently be moderately large, particularly in species with large broods. Realized genome‐wide kinship between males and dependent broods (as opposed to individual offspring) is therefore likely to be close to expectation, meaning that pedigree analysis is a useful tool with which to quantify allelic costs of actual and potential extra‐pair reproduction.

### DYNAMICS OF PATERNAL CARE, EXTRA‐PAIR REPRODUCTION, AND INBREEDING

Existing model frameworks predict that, under some circumstances, cuckolded males should reduce paternal care for their socially‐paired female's current brood if the value of future broods is expected to be higher (Westneat and Sherman 1993; Houston and McNamara 2002; Sheldon 2002; Alonzo and Klug 2012). However to date, analyses of such models have considered the relative values of current versus future broods simply as different degrees of lost paternity, and have not explicitly considered more subtle quantitative variation in kinship resulting from reproductive interactions among relatives. Our analyses emphasize that the differences in total allelic metrics between current and future broods are compound quantities, which depend not only on the relative degrees of female extra‐pair reproduction and consequent paternity loss, but also on brood sizes and on the kinship between the male and his socially‐paired females and her extra‐pair males. Four variables consequently affect the relative allelic metrics of males’ current versus future broods, even without considering the male's probability of surviving to future reproductive events, or any other dimension of variation in offspring value stemming from differential life‐history trade‐offs or environmental or maternal genetic effects (e.g., Westneat and Sherman 1993; Eliassen and Kokko 2008; Benowitz et al. 2013). Although a male's own *f* also influences allelic metrics, *f* is a fixed property of an individual male and therefore cannot contribute to within‐male variation in brood values.

The four influential variables are unlikely to be entirely under male control or readily predictable across consecutive broods. Although brood size can be influenced by males, it might commonly be primarily a female trait (Avise and Liu 2011; Kokko and Jennions 2012). Although the degree of extra‐pair paternity occurring in a brood can be influenced by both the socially‐paired female and male, female effects are larger in song sparrows (Reid et al. 2014a). A male's kinship with his current versus future socially‐paired female(s) will depend on female survival and divorce rate, on the male's kinship with new females that are available to pair, and on male and female mate choice. Meanwhile, a male's kinship with his socially‐paired female's current versus future extra‐pair males will depend on the female's extra‐pair reproductive strategy and on the availability of male relatives of the focal male, which will in turn depend on the male's previous within‐pair and extra‐pair reproductive success and that of his relatives (e.g., Reid et al. 2015b). It may therefore be difficult for individual males to “predict” the total allelic value or carrier probability of future versus current broods and modulate paternal care accordingly, unless key causal variables vary systematically with predictable aspects of male state such as age (e.g., Benowitz et al. 2013). Future analyses should quantify the pattern and magnitude of variation in total allelic metrics across consecutive broods reared by individual males in song sparrows and other systems.

The Mandarte song sparrow study population is simply one part of a large metapopulation of a predominantly sedentary species. However some dispersal does occur; Mandarte regularly receives immigrants that prevent mean *f* and *k* values from escalating (Smith et al. 2006; Wolak and Reid 2016). Similar patterns of variation in *f*_i_, *k*_ij_, and *k*_jq_ may consequently be commonplace in other spatially‐structured or primarily sedentary populations with limited dispersal. Indeed, it is increasingly clear that some degree of kin structure, and hence potential for inbreeding, occurs in diverse systems (e.g., Shorey et al. 2000; Thünken et al. 2007; reviewed by Hatchwell 2010a,b). Furthermore, females are widely hypothesized to undertake extra‐pair reproduction to avoid inbreeding, but empirical evidence suggests that they do not always avoid inbreeding through either initial pairing or extra‐pair reproduction (reviewed in Kempenaers 2007; Szulkin et al. 2013; Arct et al. 2015; Reid et al. 2015a). Together, these statements imply that socially‐paired females and males must commonly be related, and that other male relatives of the females (and hence of paired males) must commonly be available for extra‐pair mating. Logically, kinship between males and their socially‐paired females’ EPO must then sometimes exceed zero.

Perhaps unsurprisingly, experimental and correlative studies quantifying within‐population relationships between paternity and paternal care report diverse results (Sheldon 2002; Alonzo 2010; Alonzo and Klug 2012; Griffin et al. 2013). Such variation might reflect numerous causes (and biases), and a clear a priori framework of expectations has been lacking (Eliassen and Kokko 2008; Alonzo 2010). Recent comparative analyses showed that variation in the decrease in paternal care following paternity loss was associated with variation in estimated costs and benefits of care, thereby explicitly considering paternal care within the framework of kin selection theory (i.e., Hamilton's rule, Griffin et al. 2013, see also Westneat and Sherman 1993). Given this well‐established framework, it is even more surprising that quantitative variation in kinship and relatedness between interacting males and females, and hence between males and their socially‐paired females’ WPO and EPO, has not yet been explicitly considered by theoretical or empirical analyses of within‐ or among‐population variation in effects of paternity loss on paternal care in socially monogamous systems. By contrast, quantitative variation in relatedness is central to theory explaining cooperative breeding, where additional nonparent adults care for dependent young (Michod and Hamilton 1980; Hatchwell 2010b, but see Riehl 2013). Indeed, helper contributions can increase with increasing relatedness (Griffin and West 2003; Nam et al. 2010), and cooperative breeding is associated with low promiscuity (and hence high within‐family relatedness) across species (Cornwallis et al. 2010). Paternal care for unrelated EPO has also been suggested to evolve if EPO help rear subsequent WPO (Liedtke and Fromhage 2012). Furthermore, male–male relatedness might also facilitate evolution of reproductive strategies involving leks and male coalitions (Shorey et al. 2000; Krakauer 2005; Hatchwell 2010a). Further conceptual integration across different reproductive systems could be achieved by explicitly applying kin selection theory, parameterized in terms of quantitative variation in relatedness, to socially monogamous systems where cuckolded males could help their socially‐paired female rear EPO to which the male might be related through maternal and/or paternal links, thereby explicitly treating paternal care following cuckoldry as a form of kin‐selected cooperation.

New analyses of existing model frameworks, and new models, are clearly required to predict the coevolutionary dynamics of parental care and extra‐pair reproduction given variable inbreeding and kinship, thereby uniting the dual hypotheses that extra‐pair reproduction might decrease paternal care and hence experience negative selection (e.g., Westneat and Sherman 1993; Kokko 1999; Arnqvist and Kirkpatrick 2005), but might alleviate inbreeding between related socially‐paired mates and hence experience positive selection (e.g., Jennions and Petrie 2000; Kempenaers 2007). Such modeling should consider that the degrees of inbreeding expressed through social pairing and extra‐pair reproduction might themselves evolve (Reid et al. 2015c; Wolak and Reid 2016). Indeed, the hypothesis that extra‐pair reproduction allows females to avoid inbreeding does not itself explain why females pair with relatives in the first place. Such pairings might arise if there are ecological or genetic benefits of inbreeding, or costs of inbreeding avoidance (Kokko and Ots 2006; Duthie and Reid 2015; Reid et al. 2015b). However it has not been considered that pairing with a relative might reduce selection for decreased paternal care following cuckoldry, thereby creating coevolutionary feedbacks between initial mate choice and extra‐pair reproduction. Meanwhile, biparental care might increase selection against inbreeding (Kokko and Ots 2006; Duthie and Reid 2015), but female–male conflict over care might decrease following inbreeding (Thünken et al. 2007). Moreover, parental care can ameliorate inbreeding depression in offspring fitness (Pilakouta et al. 2015), and the dynamics of care might depend on parental *f*, potentially further affecting offspring state (e.g., Reid et al. 2003; Pooley et al. 2014; Mattey and Smiseth 2015). Future modeling will, therefore, need to incorporate complex direct and intergenerational effects of inbreeding on parental care and offspring fitness, alongside other forms of direct and indirect selection, to predict the coevolutionary dynamics of care, extra‐pair reproduction, and inbreeding.

## Supporting information


**Appendix S1**. Allelic metrics for within‐pair offspring.
**Appendix S2**. Allelic metrics for extra‐pair offspring.
**Appendix S3**. Supplementary figures.
