# Supplementary material for: Variation in parent–offspring kinship in socially monogamous systems with extra‐pair reproduction and inbreeding
Source: Evolution. 2016 Jun 1;70(7):1512–29. doi: 10.1111/evo.12953 (PMC4949684; doi:10.1111/evo.12953)

**Supporting Information**

**Variation in parent-offspring kinship in socially monogamous systems with extra-pair reproduction and inbreeding**

**Jane M. Reid, Greta Bocedi, Pirmin Nietlisbach, A. Bradley Duthie, Matthew E. Wolak, Elizabeth A. Gow and Peter Arcese**

**Appendix S1. Allelic metrics for within-pair offspring.**

Summary of derivations of the number of copies of an autosomal allele that is present in a focal parent that is expected to be present identical-by-descent in its within-pair offspring.

Consider a focal individual i who mates with individual j to produce a within-pair offspring (WPO) x.


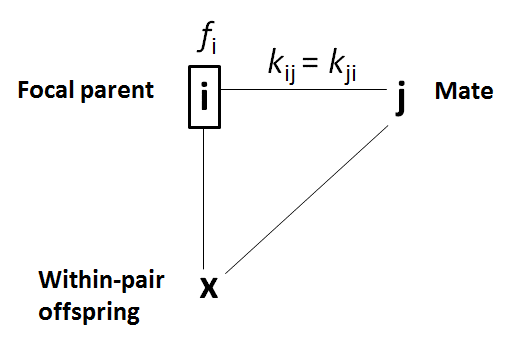


For any focal parent i:

*k*_ij_ is the coefficient of kinship between i and its mate j.

*f*_i_ is individual i’s own coefficient of inbreeding.

The probability that a WPO x will carry zero copies of an autosomal allele that is present in i is:

**P(a_ix_=0) = 0.5(1-*f*_i_)(1-*k*_ij_)**

The probability that a WPO x will carry exactly one copy of an autosomal allele that is present in i is:

P(a_ix_=1) = 0.5(1-*k*_ij_) + 0.5(*f*_i_)(1-*k*_ij_) + 0.5(1-*f*_i_)(*k*_ij_)

**P(a_ix_=1) = 0.5+0.5*f*_i_-*f*_i_*k*_ij_**

The probability that a WPO x will carry two copies of an autosomal allele that is present in i is:

**P(a_ix_=2) = 0.5*k*_ij_(1+*f*_i_)**

Hence P(a_ix_=0) + P(a_ix_=1) + P(a_ix_=2) = 1 as required.

The probability that a WPO x will carry at least one copy of an autosomal allele that is present in i (i.e. x’s ‘carrier probability’) is:

P(C_ix_) = 0.5+0.5*f*_i_-*f*_i_*k*_ij_ + 0.5*k*_ij_(1+*f*_i_)

**P(C_ix_) = 0.5(1+*f*_i_+*k*_ij_-*f*_i_*k*_ij_)**

Hence P(C_ix_) = 1 - P(a_ix_=0) as required.

The number of copies of an autosomal allele that is present in i that is expected to be present identical-by-descent in a WPO x is therefore:

E(a_ix_) = 0(0.5(1-*f*_i_)(1-*k*_ij_) + 1(0.5+0.5*f*_i_-*f*_i_*k*_ij_) + 2(0.5*k*_ij_(1+*f*_i_))

**E(a_ix_) = 0.5+0.5*f*_i_+*k*_ij_**

This expression has previously been presented (e.g. Falconer and Mackay 1996, p.87; Lynch and Walsh 1998, eqn 7.4).

The expected number of copies of an allele that is present in i that will be present in a WPO x per copy in i is therefore:

**E(a_ix_|a_i_) = (0.5+0.5*f*_i_+*k*_ij_)/(1+*f*_i_)**

The sampling variance in the number of copies of an autosomal allele that is present in i that will be present in a WPO x is given by the general definition var(χ) = E(χ^2^) - E(χ)^2^, where χ is any variable. Hence:

var(a_ix_) = E(a_ix_^2^) - E(a_ix_)^2^

var(a_ix_) = (0.5+0.5*f*_i_-*f*_i_*k*_ij_) + 4(0.5*k*_ij_(1+*f*_i_)) – (0.5+0.5*f*_i_+*k*_ij_) (0.5+0.5*f*_i_+*k*_ij_)

**var(a_ix_) = 0.25+*k*_ij_-0.25*f*_i_^2^-*k*_ij_^2^**

**Appendix S2. Allelic metrics for extra-pair offspring.**

Summary of derivations of the number of copies of an autosomal allele that is present in a focal socially-paired male that is expected to be present identical-by-descent in the extra-pair offspring that it rears.

Consider a focal male j that is socially-paired with female i, who then mates with extra-pair male q to produce an extra-pair offspring (EPO) y.


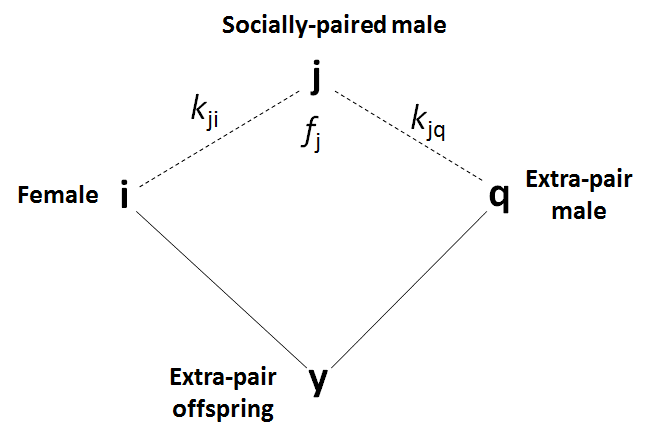


For any individual j:

*k*_ji_ is the coefficient of kinship between male j and its socially-paired female i.

*k*_jq_ is the coefficient of kinship between male j and its socially-paired female’s extra-pair male q.

*f*_j_ is individual j’s own coefficient of inbreeding.

The probability that an EPO y will carry zero copies of an autosomal allele that is present in j is:

P(a_jy_=0) = (1-*k*_ji_)(1-*k*_jq_)

**P(a_jy_=0) = 1-*k*_ji_-*k*_jq_+*k*_ji_*k*_jq_**

The probability that an EPO y will carry exactly one copy of an autosomal allele that is present in j is:

P(a_jy_=1) = *k*_ji_(1-*k*_jq_) + *k*_jq_(1-*k*_ji_)

**P(a_jy_=1) = *k*_ji_+*k*_jq_-2*k*_ji_*k*_jq_**

The probability that an EPO y will carry two copies of an autosomal allele that is present in j is:

**P(a_jy_=2) = *k*_ji_*k*_jq_**

Hence P(a_jy_=0) + P(a_jy_=1) + P(a_jy_=2) = 1 as required.

The probability that an EPO y will carry at least one copy of an autosomal allele that is present in j (i.e. y’s ‘carrier probability’) is:

P(C_jy_) = P(a_jy_=1) + P(a_jy_=2)

**P(C_jy_) = *k*_ji_+*k*_jq_-*k*_ji_*k*_jq_**

Hence P(C_jy_) = 1 - P(a_jy_=0) as required.

The expected number of copies of an autosomal allele that is present in j that will be present identical-by-descent in an EPO y is therefore:

E(a_jy_) = P(a_jy_=1) + 2P(a_jy_=2)

**E(a_jy_) = *k*_jy_+*k*_jq_**

The expected number of copies of an allele that is present in j that will be present in an EPO y per copy in j is therefore:

**E(a_jy_|a_j_) = (*k*_iq_+*k*_jq_)/(1+*f*_j_)**

The sampling variance in the number of copies of an allele that is present in j that will be present in an EPO y is:

var(a_jy_) = E(a_jy_^2^) - E(a_jy_)^2^

var(a_jy_) = *k*_ji_+*k*_jq_-2*k*_ji_*k*_jq_ + 4*k*_ji_*k*_jq_ – (*k*_jy_+*k*_jq_)^2^

var(a_jy_) = *k*_ji_+*k*_jq_-2*k*_ji_*k*_jq_ + 4*k*_ji_*k*_jq_ – (*k*_jy_^2^+*k*_jq_^2^+2*k*_jy_*k*_jq_)

**var(a_jy_) = *k*_ji_+*k*_jq_-*k*_ji_^2^-*k*_jq_^2^**

**Appendix S3. Supplementary figures.**

**Figure S1.** Distributions of (A and B) carrier probabilities P(C_ix_), (C and D) allelic values per copy in parent i E(a_ix_|a_i_) and (E and F) allelic variances var(a_ix_) of 1526 observed within-pair offspring (WPO) relative to their (A, C and E) mother and their (B, D and F) father (i.e. their mother’s socially-paired male).


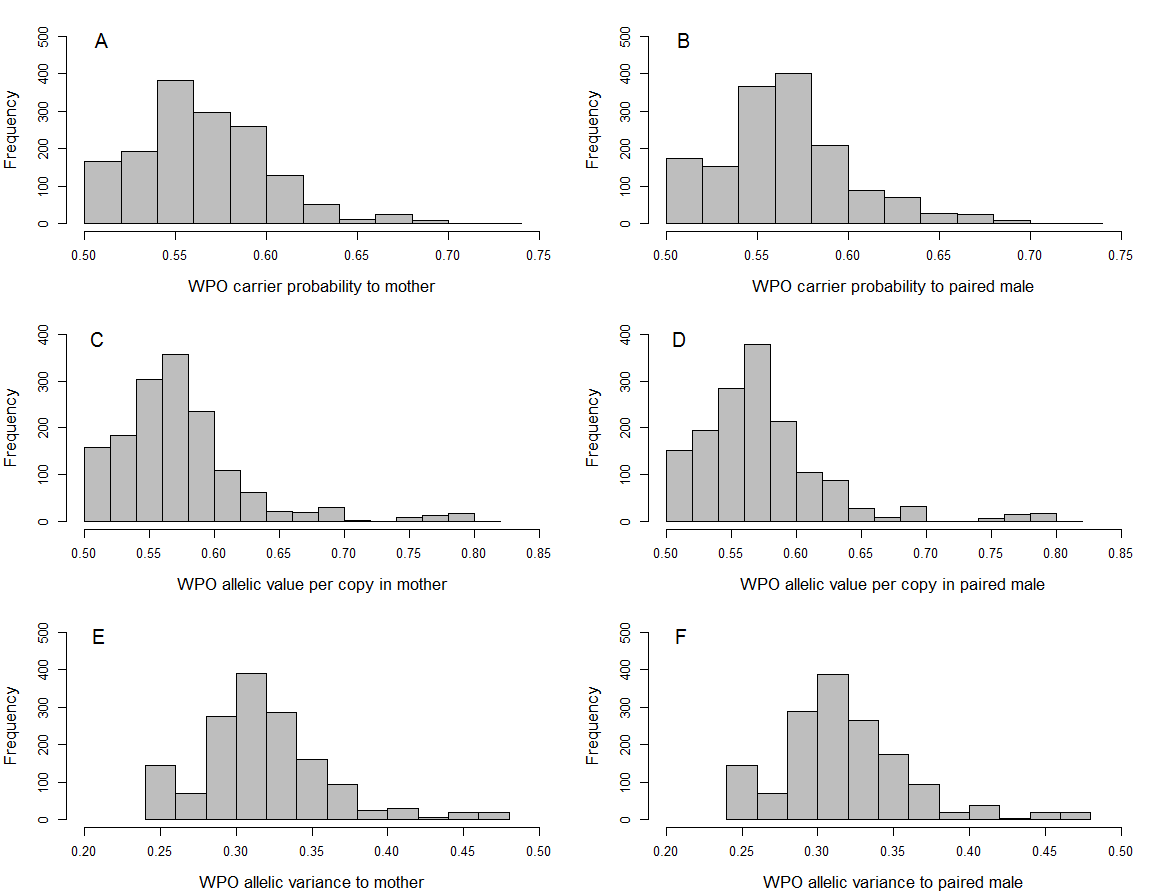


**Figure S2.** Relationships between the (A) allelic values per copy in parent i E(a_ix_|a_i_) and (B) allelic variances var(a_ix_) of 1526 observed within-pair offspring (WPO) relative to their mother versus father (i.e. the mother’s socially-paired male). Lines depict linear regressions. Pearson correlations between WPOs’ values relative to their two parents are both ca. 0.99.


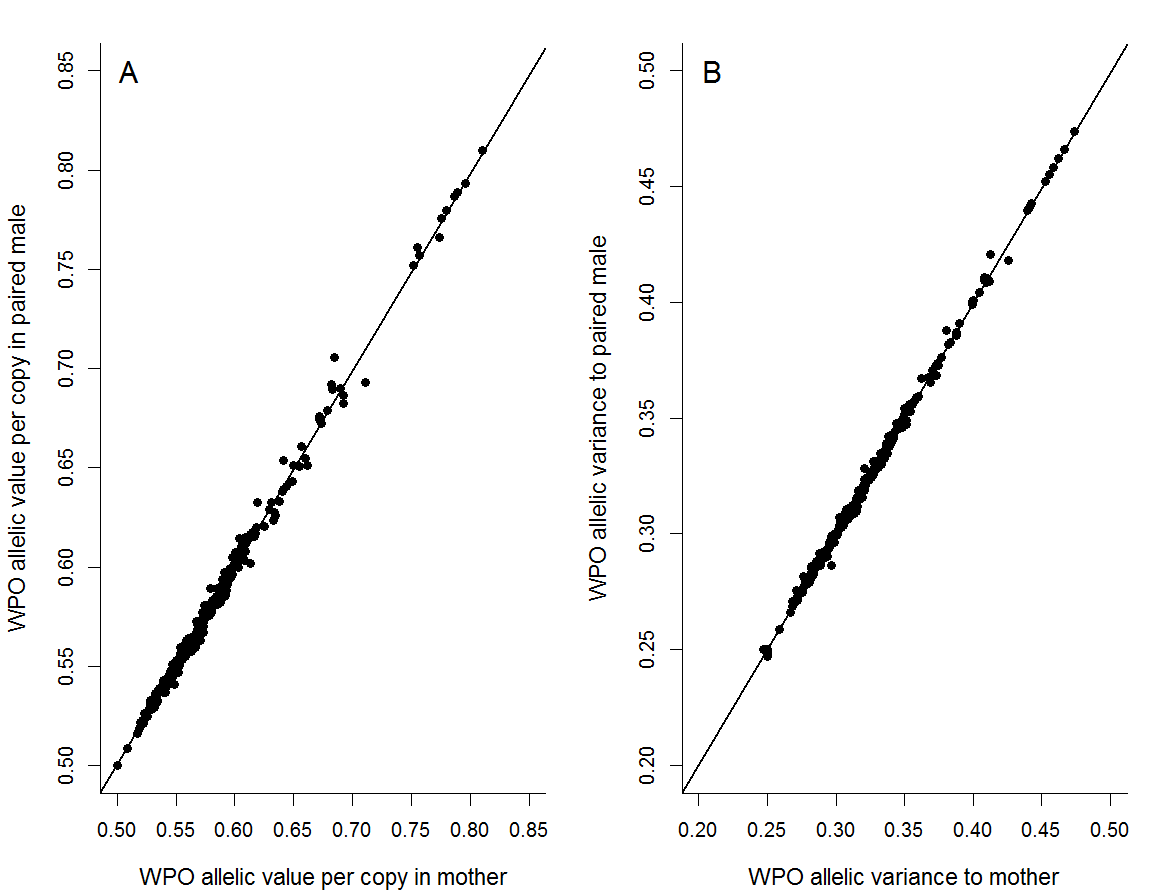


**Figure S3.** Distributions of (A) carrier probability P(C_jy_), (B) allelic value per copy E(a_jy_|a_j_) and (C) allelic variance var(a_jy_) of 561 observed extra-pair offspring (EPO) relative to their mother’s socially-paired male, and of the difference in (D) carrier probability, (E) allelic value per copy and (F) allelic variance between the male’s potential within-pair offspring and its observed extra-pair offspring.


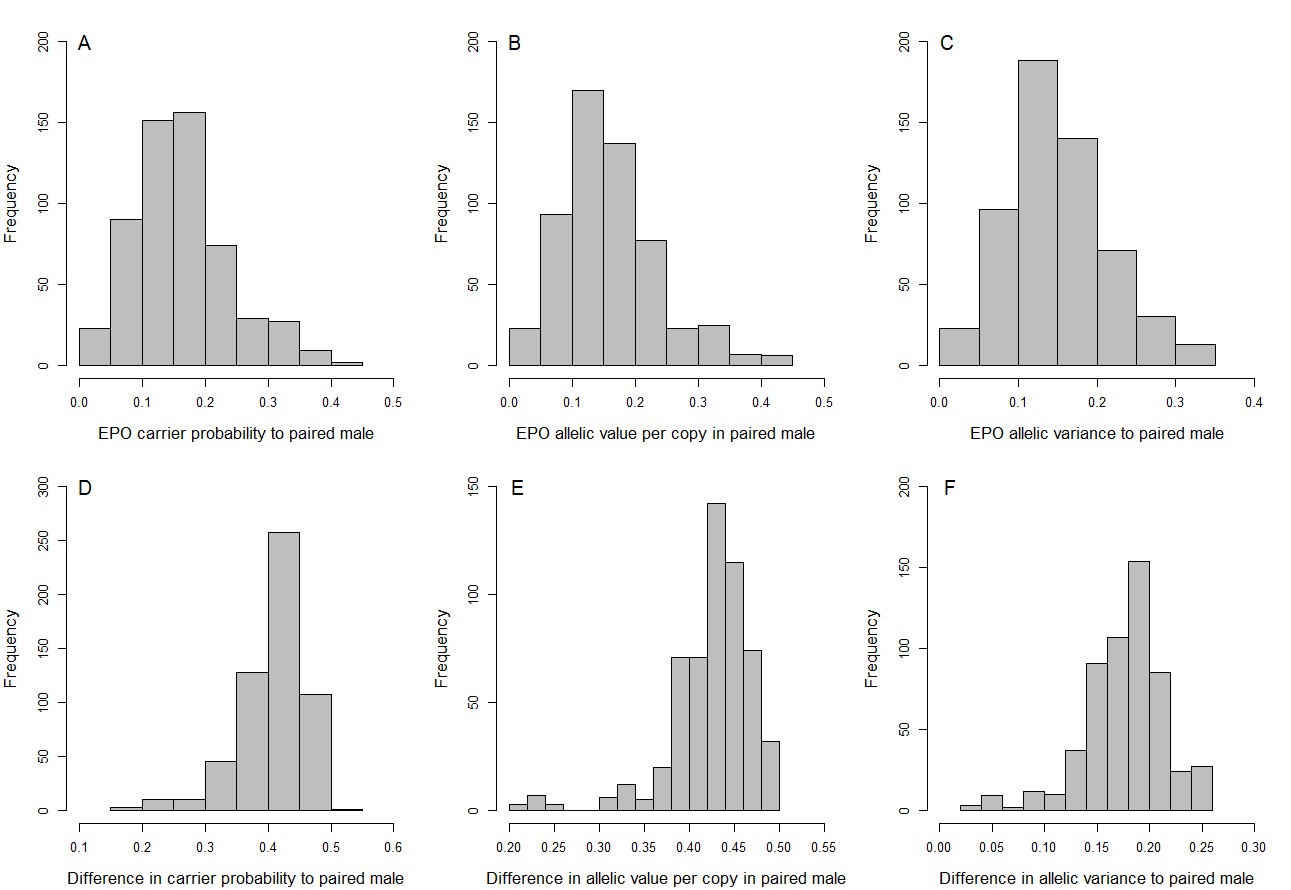


**Figure S4.** Relationships between the difference in (A-C) allelic value E(a), (D-F) carrier probability P(C), (G-I) allelic value per copy E(a|j) and (J-L) allelic variance var(a) between a socially-paired male’s observed extra-pair offspring (EPO) and the within-pair offspring (WPO) that the male could potentially have sired and (A, D, G and J) the male’s kinship with his socially-paired female *k*_ji_, (B, E, H and K) the male’s kinship with his socially-paired female’s extra-pair male *k*_jq_, and (C, F, I and L) the male’s own coefficient of inbreeding *f*_j_, across 561 observed EPO.


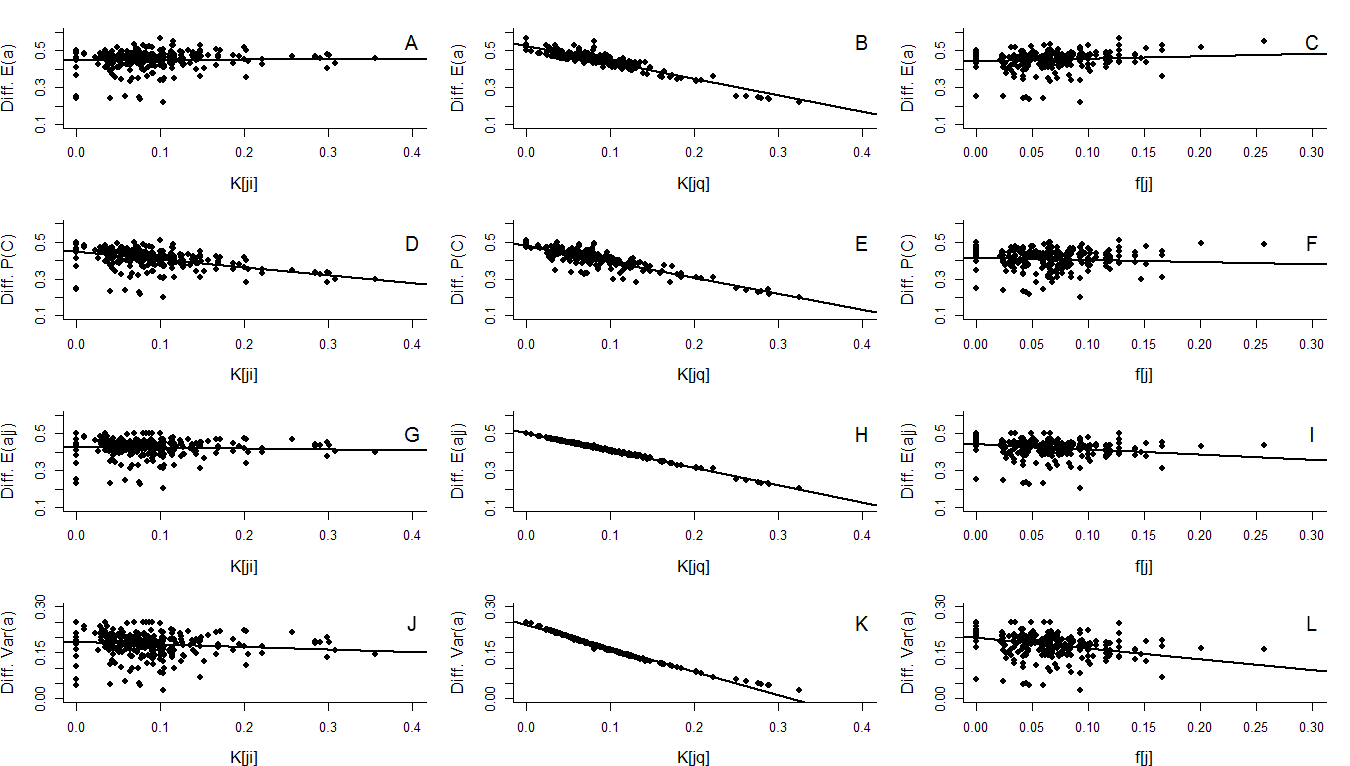


**Figure S5.** Relationships between the (A) carrier probability, (B) allelic value per copy and (C) allelic variance between 561 observed extra-pair offspring (EPO) produced by males’ socially-paired females, and the alternative within-pair offspring (WPO) that the males could potentially have sired.


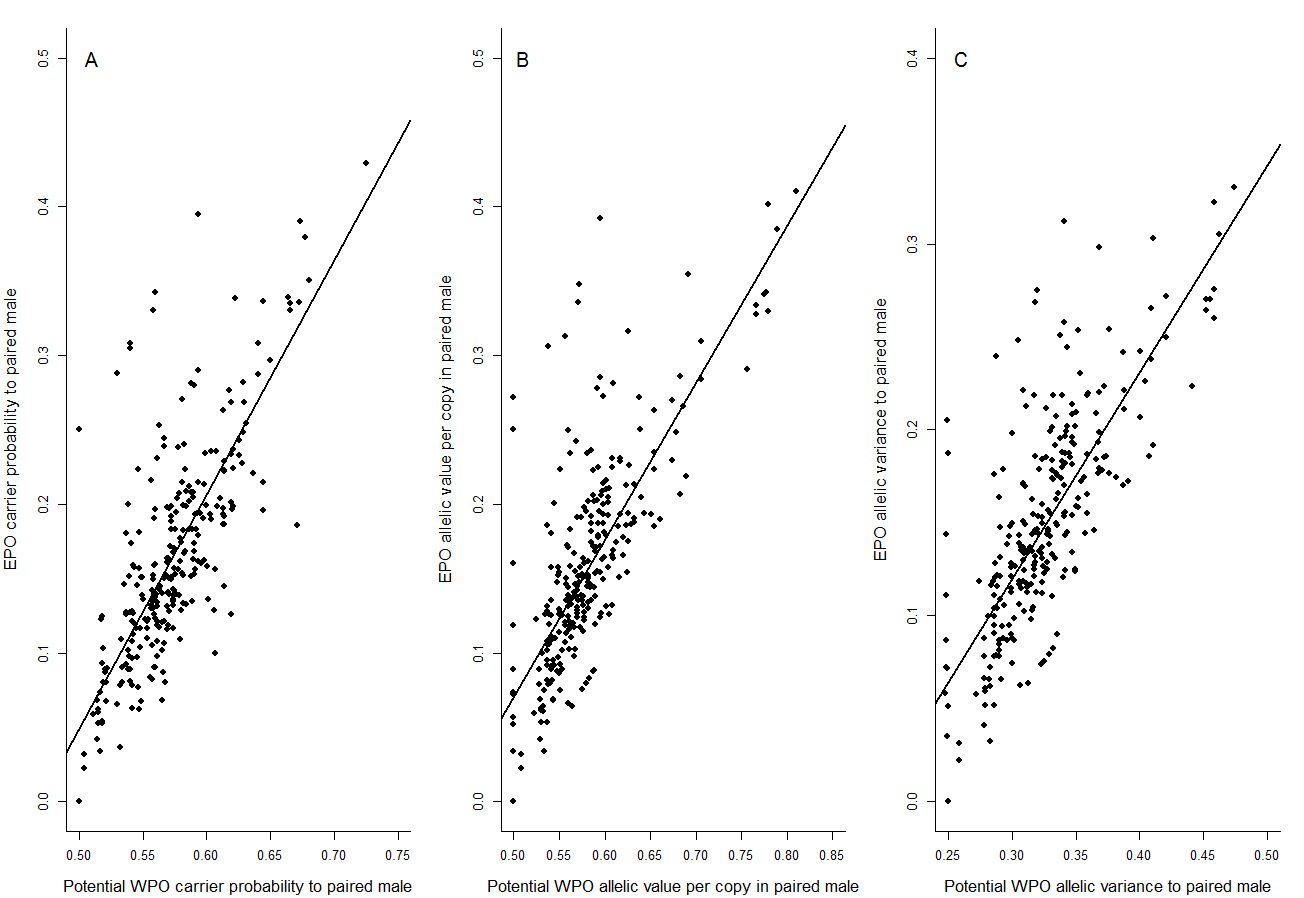


**Figure S6.** Distributions of (A) carrier probability, (B) allelic value per copy and (C) allelic variance of 561 observed extra-pair offspring (EPO) relative to their mother, and of the difference in (D) carrier probability, (E) allelic value per copy and (F) allelic variance between these observed EPO and the alternative within-pair offspring (WPO) that their mother could have produced with her socially-paired male.


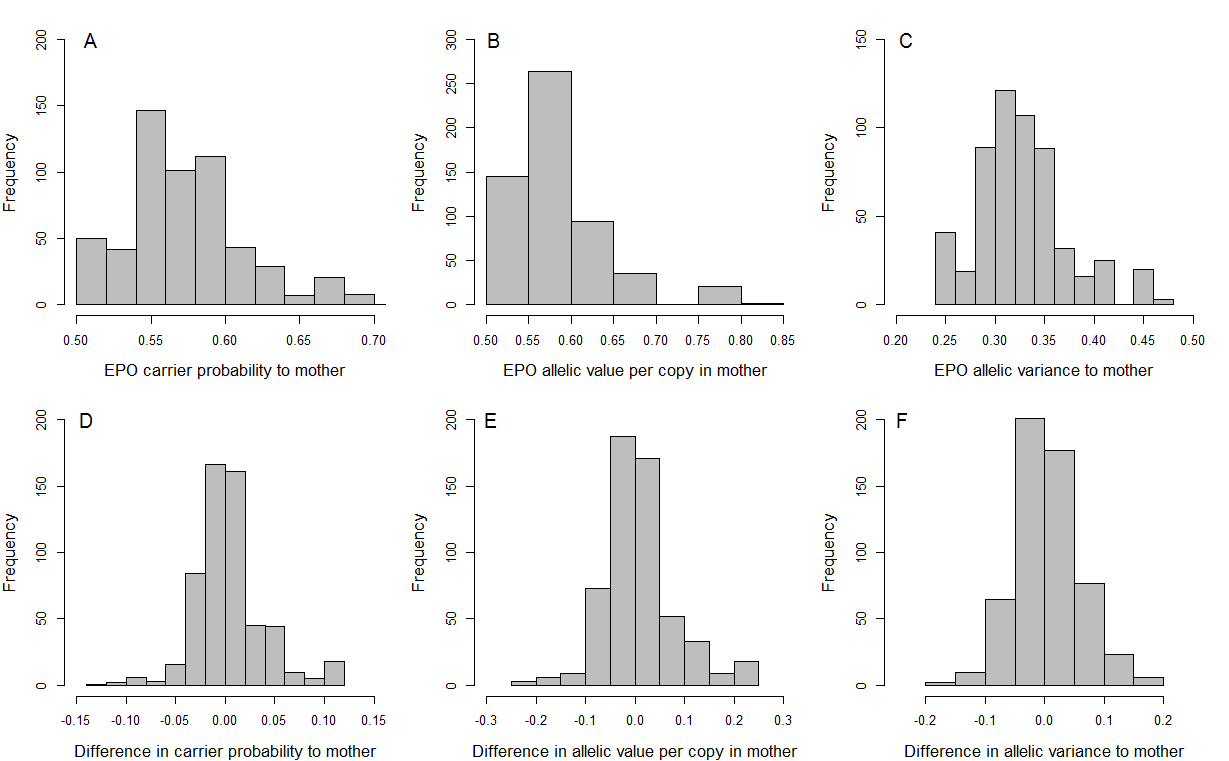


**Figure S7.** Distributions of the difference in (A) carrier probability, (B) allelic value per copy and (C) allelic variance of 561 observed extra-pair offspring (EPO) relative to their mother versus her socially-paired male.


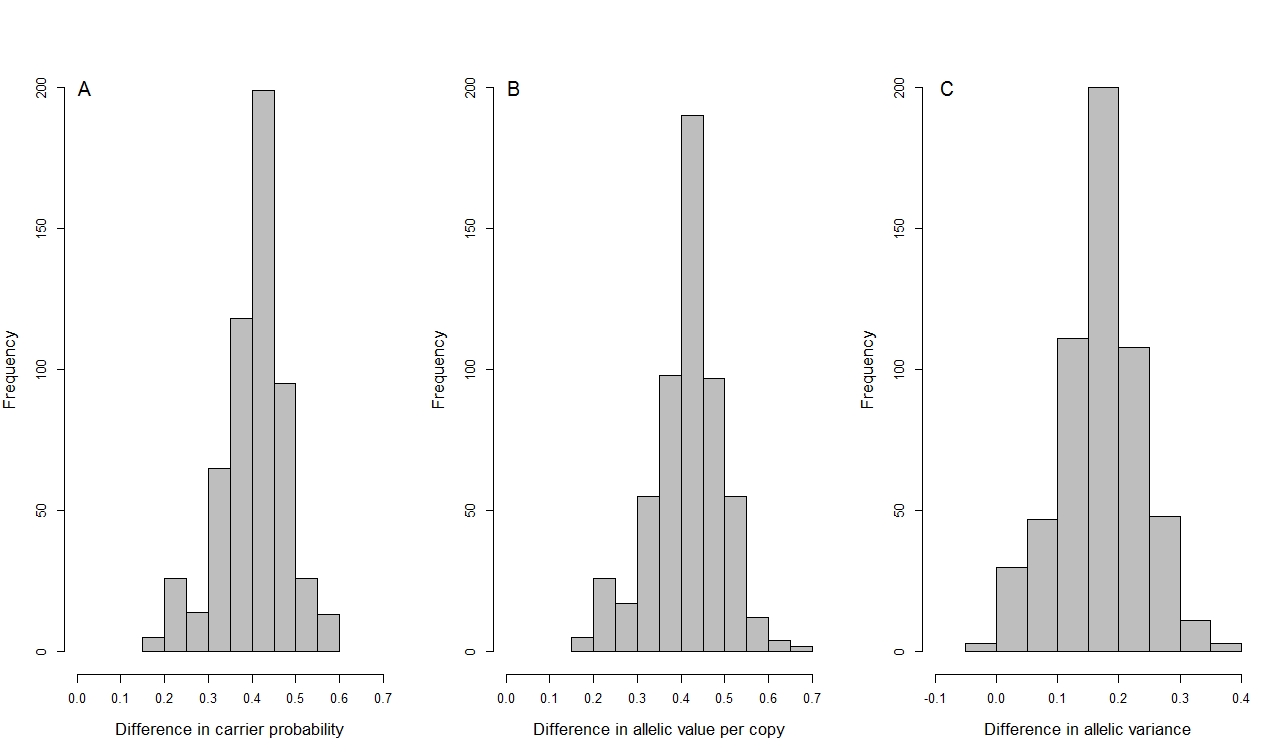


**Figure S8.** Relationships between the (A) carrier probability, (B) allelic value per copy and (C) allelic variance of 561 observed extra-pair offspring (EPO) relative to their mother versus her socially-paired male, and between the difference in (D and G) carrier probability, (E and H) allelic value per copy and (F and I) allelic variance relative to an EPO’s mother versus her socially-paired male and the coefficient of kinship between (D-F) the socially-paired female and male (k_ji_) and (H-I) the female’s socially-paired male and extra-pair male (k_jq_).


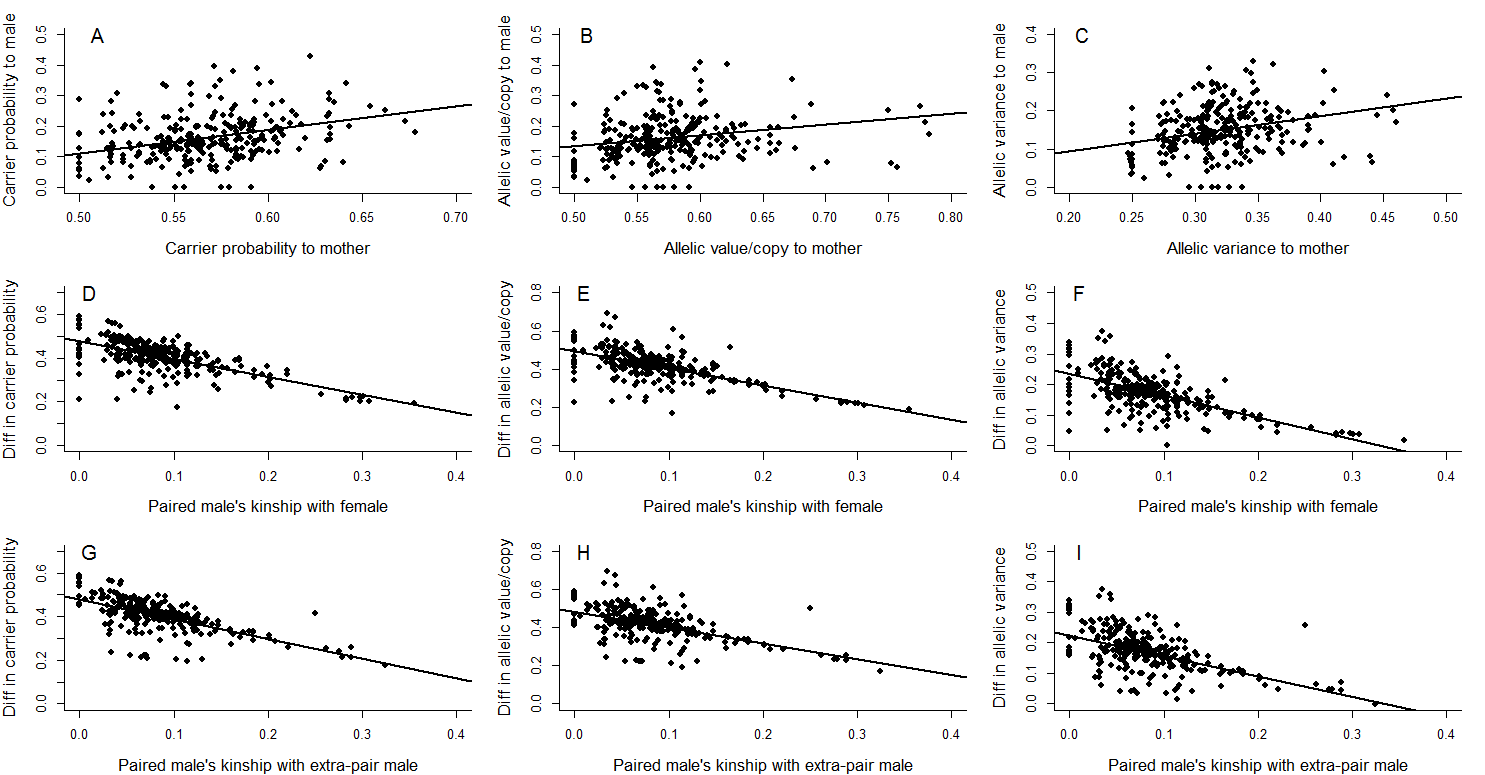


**Figure S9.** Relationships between brood size and the brood’s (A and B) total allelic value E(a), (C and D) total carrier probability P(C), (E and F) total allelic value per copy in the focal adult E(a|i) or E(a|j) and (G and H) total allelic variance Var(a) relative to its (A, C, E and G) mother and (B, D, F and H) its mother’s socially-paired male, across 741 observed broods. Pearson correlation coefficients between brood size and allelic metrics were (A) 0.95, (C) 0.91, (E) 0.97 and (G) 0.93 for mothers and (B) 0.69, (D) 0.55, (F) 0.70 and (H) 0.76 for socially-paired males.


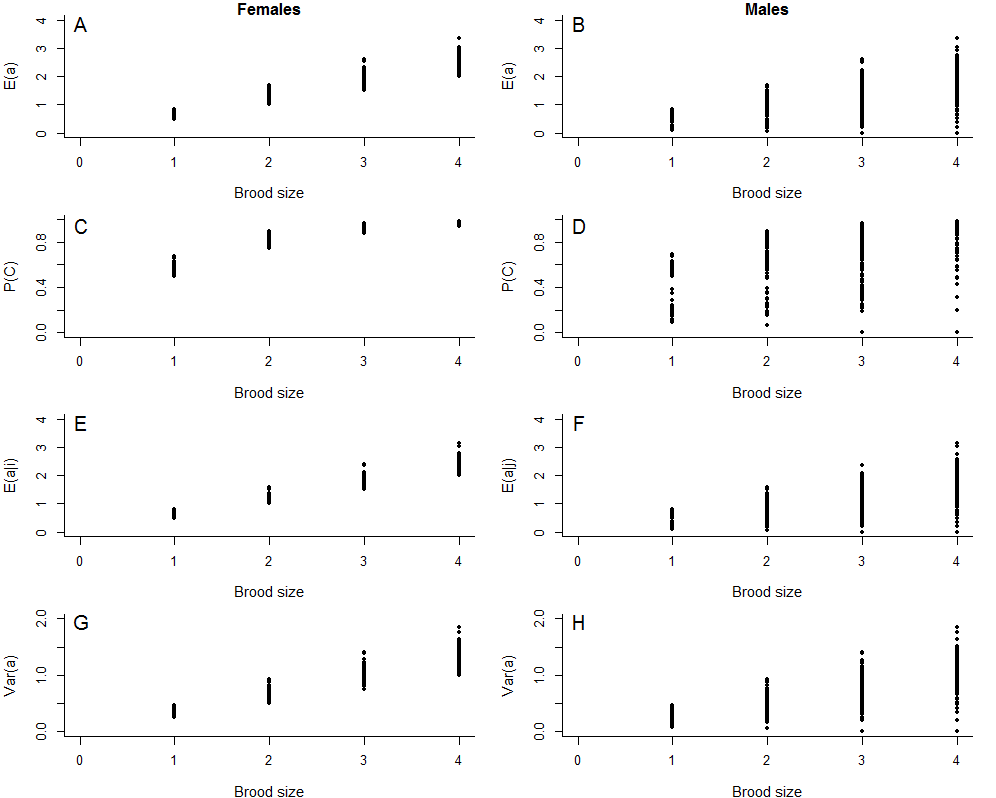


**Figure S10.** Distributions of the differences in a brood’s total (A) allelic value, (B) carrier probability, (C) allelic value per copy in the focal adult and (D) allelic variance relative to the brood’s mother versus her socially-paired male, across 420 observed broods that only contained within-pair offspring (WPO-broods).


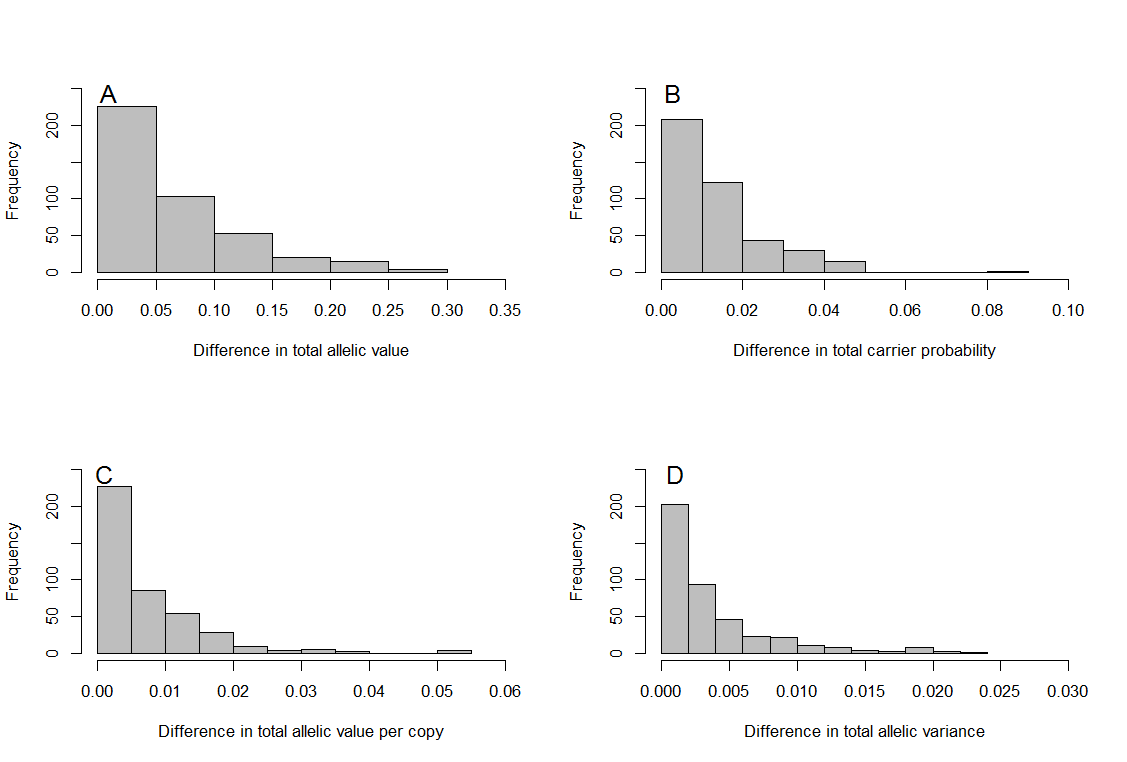


**Figure S11.** Distributions of the differences in a brood’s total (A) allelic value, (B) carrier probability, (C) allelic value per copy in the focal adult and (D) allelic variance relative to the brood’s mother versus her socially-paired male, across 321 observed broods that contained at least one extra-pair offspring (EPO-broods).


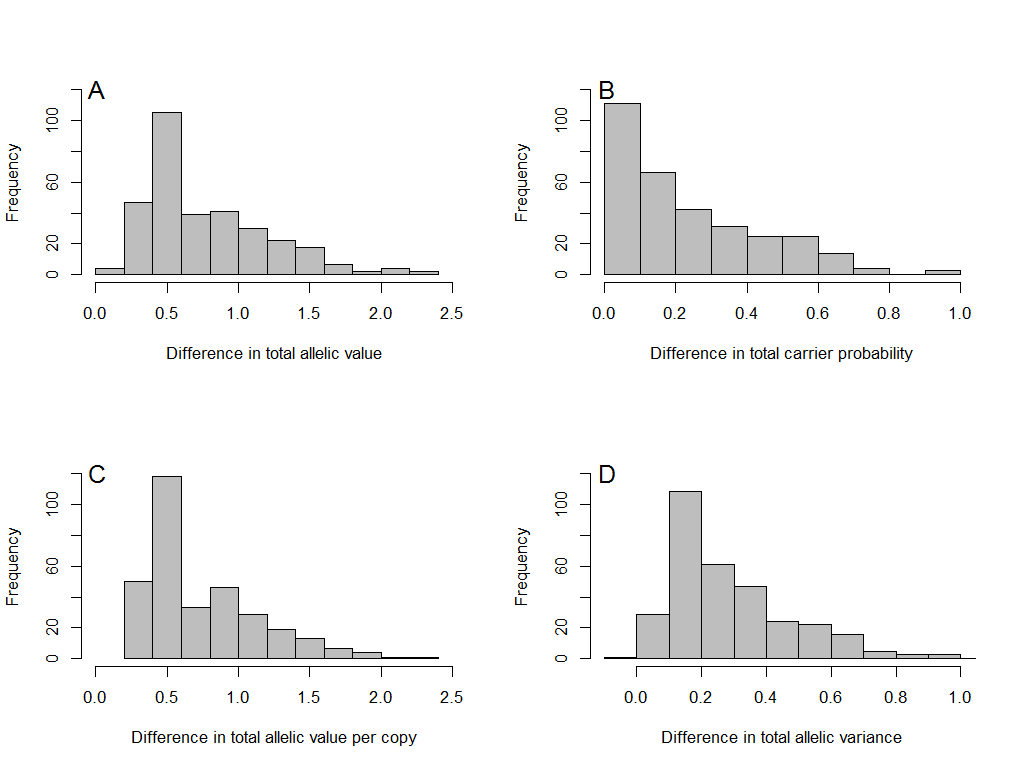


**Figure S12.** Distributions of the absolute decrease in total (A) allelic value, (B) carrier probability, (C) allelic value per copy in the focal male and (D) allelic variance of socially-paired male’s potential WPO-broods versus their observed EPO-broods, across 321 observed EPO-broods.


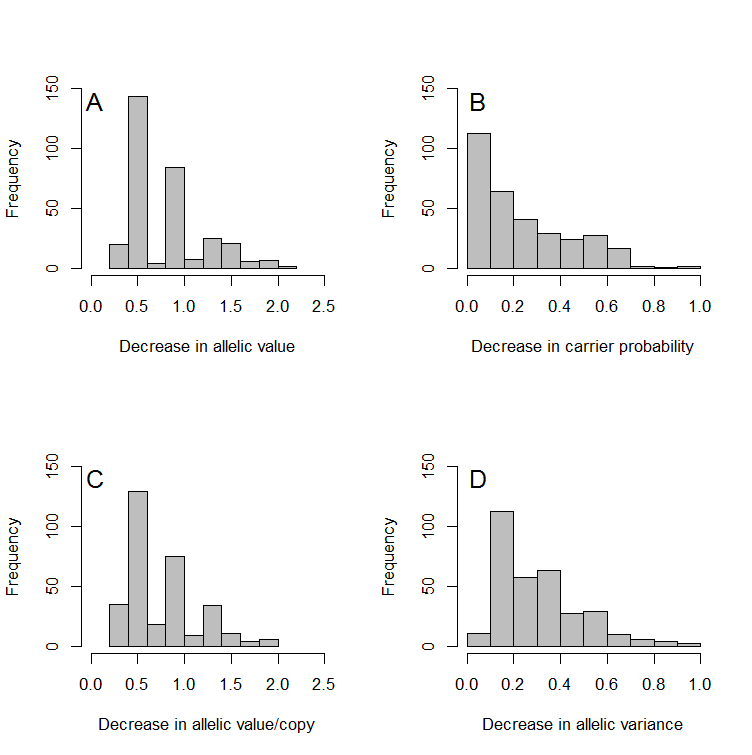


**Figure S13.** Distributions of the proportional decrease in total (A) allelic value, (B) carrier probability, (C) allelic value per copy in the focal male and (D) allelic variance of socially-paired male’s potential WPO-broods versus their observed EPO-broods, across 321 observed EPO-broods, calculated relative to the values of the potential WPO-broods.


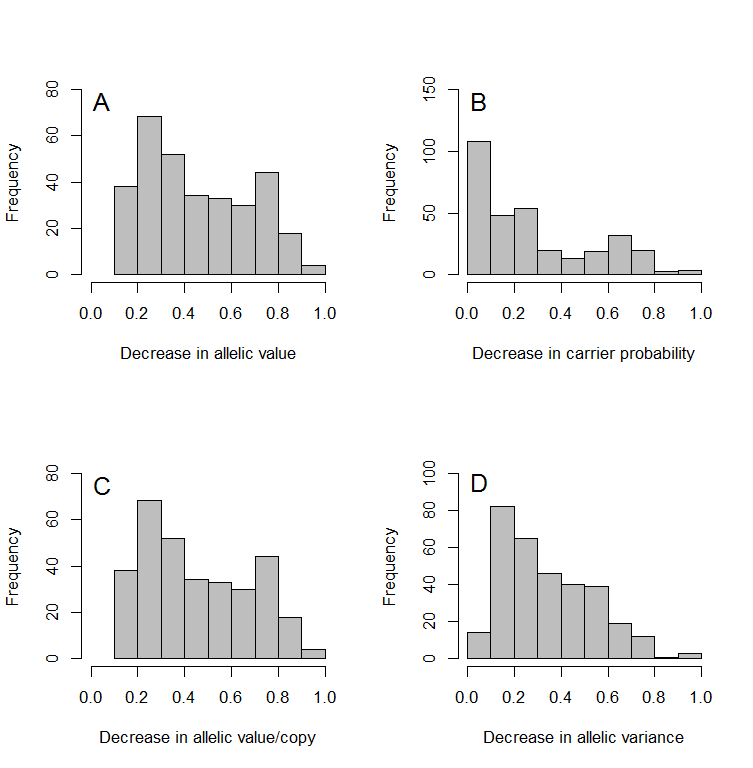


**Figure S14.** Distributions of the (A and B) absolute and (C and D) proportional decreases in total brood (A and B) allelic value and (C and D) allelic variance of socially-paired male’s potential WPO-broods versus their observed EPO-broods, across 321 observed EPO-broods given no inbreeding (i.e. *f*_i_=0, *k*_ji_=0 and *k*_jq_=0). Proportional values were calculated relative to the values of the potential WPO-broods. Values for total brood carrier probability and allelic value per copy in the focal male are the same as those for total brood allelic value.


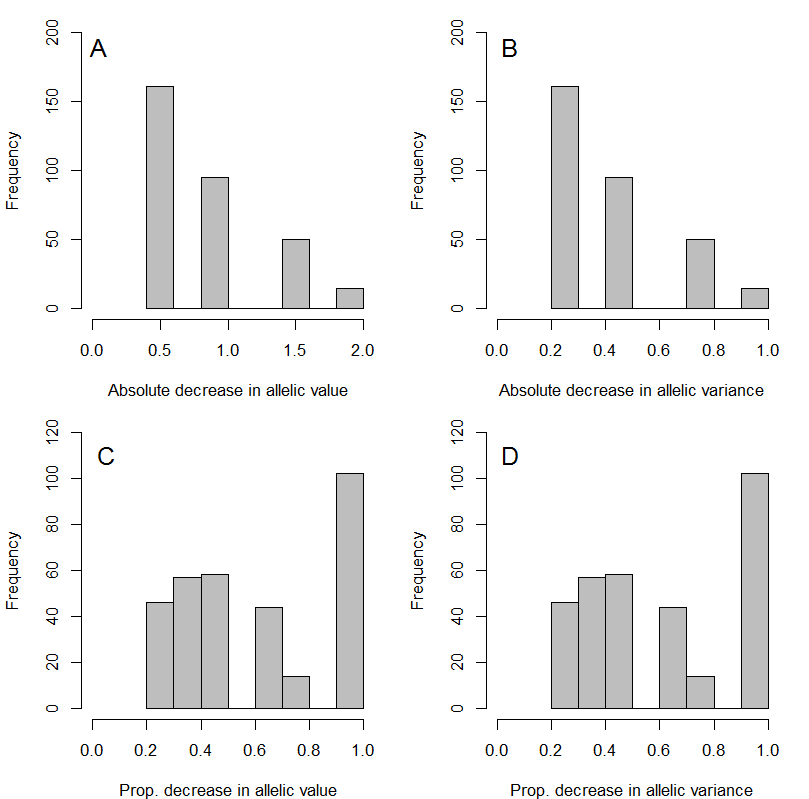


**Figure S15.** Relationship between a brood’s total allelic value and allelic variance relative to its mother’s socially-paired male across 741 observed broods. Points and triangles denote observed WPO-broods (N = 420) and EPO-broods (N = 321) respectively. Black, dark-grey, mid-grey and light-grey symbols denote brood sizes of 4, 3, 2 and 1 chick respectively. Dotted and dashed lines depict linear regressions fitted through WPO-broods and EPO-broods respectively. Allelic variances are slightly higher for EPO-broods than for WPO-broods given the same allelic value because EPO-broods contain more chicks than WPO-broods for the same allelic value, and hence experience more allele sampling events.


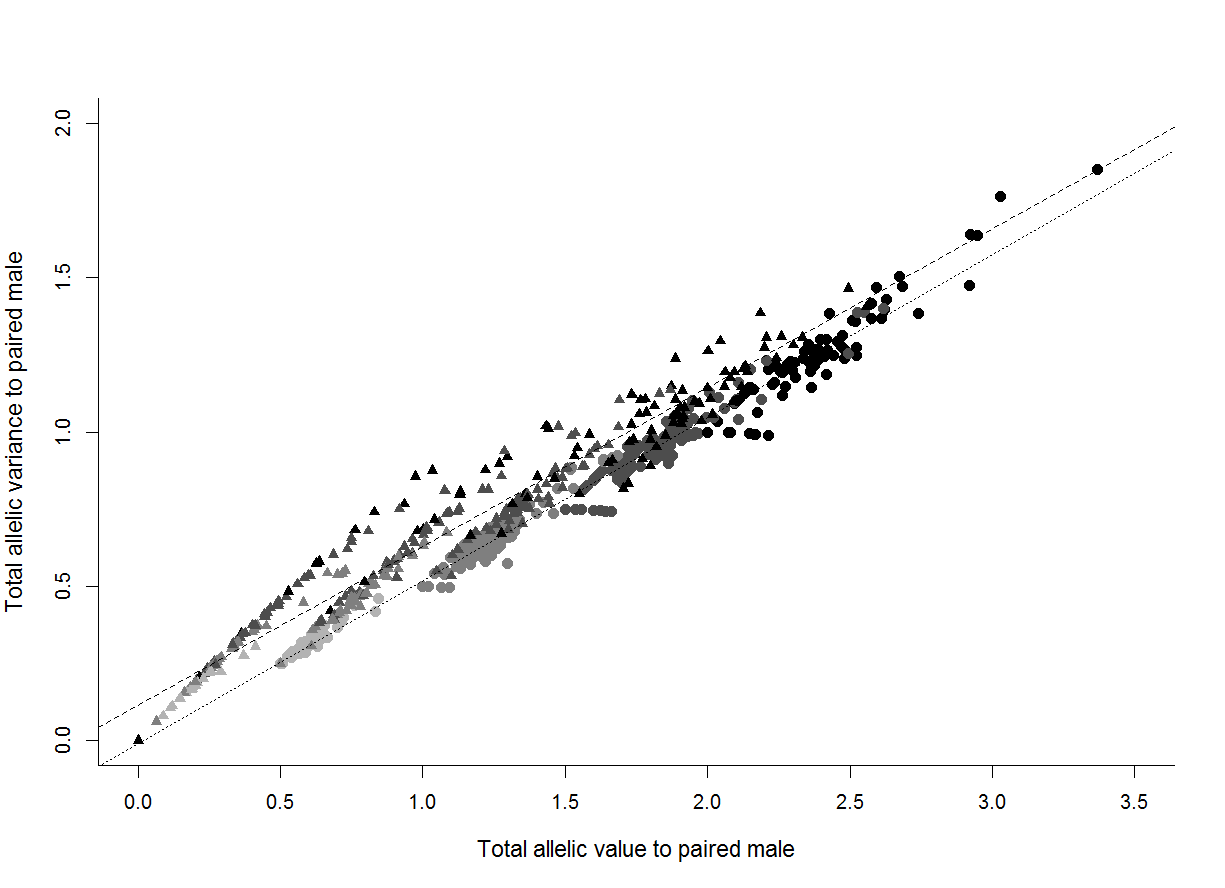

Supplement: Supplementary file 1 — Appendix S1. Allelic metrics for within‐pair offspring. Appendix S2. Allelic metrics for extra‐pair offspring. Appendix S3. Supplementary figures. [file EVO-70-1512-s001.docx]
